# Graphical Models in Reconstructability Analysis and Bayesian Networks

**DOI:** 10.3390/e23080986

**Published:** 2021-07-30

**Authors:** Marcus Harris, Martin Zwick

**Affiliations:** Systems Science Program, Portland State University, Portland, OR 97207, USA; zwick@pdx.edu

**Keywords:** probabilistic graphical models, Reconstructability Analysis, Bayesian networks, information theory, maximum entropy, artificial intelligence, machine learning, lattice of general structures, hypergraph, directed acyclic graph

## Abstract

Reconstructability Analysis (RA) and Bayesian Networks (BN) are both probabilistic graphical modeling methodologies used in machine learning and artificial intelligence. There are RA models that are statistically equivalent to BN models and there are also models unique to RA and models unique to BN. The primary goal of this paper is to unify these two methodologies via a lattice of structures that offers an expanded set of models to represent complex systems more accurately or more simply. The conceptualization of this lattice also offers a framework for additional innovations beyond what is presented here. Specifically, this paper integrates RA and BN by developing and visualizing: (1) a BN neutral system lattice of general and specific graphs, (2) a joint RA-BN neutral system lattice of general and specific graphs, (3) an augmented RA directed system lattice of prediction graphs, and (4) a BN directed system lattice of prediction graphs. Additionally, it (5) extends RA notation to encompass BN graphs and (6) offers an algorithm to search the joint RA-BN neutral system lattice to find the best representation of system structure from underlying system variables. All lattices shown in this paper are for four variables, but the theory and methodology presented in this paper are general and apply to any number of variables. These methodological innovations are contributions to machine learning and artificial intelligence and more generally to complex systems analysis. The paper also reviews some relevant prior work of others so that the innovations offered here can be understood in a self-contained way within the context of this paper.

## 1. Introduction

Reconstructability Analysis (RA) and Bayesian Networks (BN) are both probabilistic graphical modeling methodologies. A probabilistic graphical model uses a graph (or hypergraph) to encode independencies and dependencies between variables and probability theory to encode the precise nature of the relations between variables. Graphs are either undirected or directed. RA graphs include undirected graphs (or hypergraphs) that have loops or do not have loops. BN graphs are directed graphs that do not have cycles. (“Loops” here refer to undirected graphs; “cycles” refer to directed graphs.) RA and BN graphs can represent independence structures that are unique to each methodology, and also independence structures that are the same in both methodologies. For RA models without loops and for all BN models, variable independencies can be represented in closed algebraic (factorized) form. For RA models with loops, solutions require iterative calculations. The value of integrating these two methodologies lies in the fact that the RA lattice of structures offers potential models of complex systems not found in BNs, while BNs are a more widely used analytical approach than RA and also include unique models. Combining the candidate models of the two methodologies thus offers a more expressive framework than either alone. It also does so in an organized and coherent way that allows for future possible extensions discussed in Section 6.

RA is a data modeling approach developed in the systems community [1,2,3,4,5,6,7,8,9,10,11,12,13,14,15,16,17] that combines graph theory and information theory. Its applications are diverse, including time-series analysis, classification, decomposition, compression, pattern recognition, prediction, control, and decision analysis [14]. It is designed especially for nominal variables, but continuous variables can be accommodated if their values are discretized. RA could in theory accommodate continuous variables; however, this extension of the methodology has yet to be formalized. Graph theory specifies the structure of the model: if the relations between the variables are all dyadic (pairwise), the structure is a graph; if some relations have higher ordinality, the structure is a hypergraph. In speaking of RA, the word ‘graph’ will henceforth include the possibility that the structure is a hypergraph. The structure is independent of the data except for specification of variable cardinalities. In RA, information theory uses the data to characterize the precise nature and the strength of the relations. Data applied to a graph structure yields a probabilistic graphical model of the data. 

RA has three primary types of models: variable-based models without loops, variable-based models with loops and state-based models (where individual states of variables specify model constraints) that nearly always have loops. Models that do not have loops have closed-form algebraic solutions; those that have loops require iterative proportional fitting. In RA, graphs are undirected, although directions are implicit if one variable is designated as the response variable (dependent variable or DV), while all other variables are designated as explanatory variables (independent variables or IVs). In principle, there could be more than one DV, but in the discussion that follows, a single DV is assumed. If the IV-DV distinction is made, the system is ‘directed’ and the primary aim is prediction of the DV given the IVs; if no IV-DV distinction is made, the system is ‘neutral’ and the primary aim is to characterize the nature of relations among all variables. 

RA models are undirected graphs that either have or do not have loops, where a ‘loop’ is the presence of circularity in a set of undirected links. We reserve the word ‘cycle’ and ‘acyclic’ for circularity or lack thereof in directed graphs, which are used in BN and not in RA. An undirected graph having a loop can become an acyclic graph for certain assignments of link directions. For example, an RA model that posits relations between A and B, between B and C, and between A and C has a loop, but if directions are assigned in a BN model so that these relations are A→B, B→C, and A→C, the resulting graph is acyclic. 

Graphs are general or specific. A general graph identifies relations among variables that are unlabeled, i.e., variables whose identity is not specified; a specific graph labels (identifies) the variables. For example, for a system consisting of variables A, B, and C, AB:BC is a specific graph where nodes A and B are linked and B and C are also linked. Specific graphs AB:BC, BA:AC and AC:CB are all instances of the same general graph that has a unique independence structure regardless of variable labels. In this notation, the order of variables in any relation is arbitrary, as is the order of the relations. For example, CB:BA is identical to AB:BC. Relations include all of their embedded relations. For example, ABC includes embedded relations AB, AC and BC and the univariate margins A, B, and C. 

The lattice of graphs for a neutral or a directed system with or without loops depends upon the number of variables in the data. For a three-variable neutral system allowing loops there are five general graphs and nine specific graphs; for four variables there are 20 general graphs and 114 specific graphs. The number of graphs increases hyper-exponentially with the number of variables. In the confirmatory mode, RA can test the significance of a single model—a hypothesis being tested—relative to another model used as a reference. In the exploratory mode, RA can search the lattice of graphs for models that are statistically significant and best represent the data with maximal information captured and minimal complexity. 

Bayesian Networks (BN) are another probabilistic graphical modeling approach to data modeling that is closely related to RA. Indeed, where BN overlaps RA the two methods are equivalent, but with respect to neutral systems, RA and BN each has distinctive features absent in the other methodology. For directed systems; however, where prediction of a single dependent variable is the aim, RA encompasses all models found in BN under the convention used in this paper that all nodes except for parent nodes within a V-structure are allowed to be the variable being predicted; this inclusion of the BN directed system lattice within the RA lattice will be shown later in this paper. 

BNs have origins in the type of path model described by Wright [18,19], but it was not until the 1980s that BNs became more formally established [20,21,22,23]. As does RA, BN combines graph theory and probability theory: graph theory provides the structure and probability theory characterizes the nature of relationships between variables. BNs are represented by a single type of graph structure; a directed acyclic graph, which is a subset of chain graphs, also known as block recursive models [24]. BNs can be represented more generally by partially directed acyclic graphs (PDAG), a subset of chain graphs where edge directions are removed when directionality has no effect on the underlying independence structure. Discrete variables are most common in BNs, but BNs accommodate continuous variables without discretization [25]. In principal RA could also accommodate continuous variables but this feature has not yet been implemented. For a three variable BN lattice, there are 5 general graphs and 11 specific graphs; for four variables there are 20 general graphs and 185 specific graphs with unique probability distributions. In the confirmatory mode, BNs can test the significance of a model relative to another model used as a reference [26]; in the exploratory mode, BNs can search for the best possible model given a scoring metric. BNs are used to model expert knowledge about uncertainty and causality [20,21] and are also used for exploratory data analysis with no use of expert knowledge [27]. Like RA, BN applications in machine learning and artificial intelligence are broad including classification, prediction, compression, pattern recognition, image processing, time-series, decision analysis and many others.

The joint RA-BN lattice of neutral system general and specific graphs and the accompanying search algorithm developed in this paper expands both RA and BN beyond what was previously available by either RA alone or BN alone, thus providing a more complete ensemble of models for the representation of complex systems. When prediction of a single dependent variable (DV) is the aim, the RA directed system lattice encompasses the BN directed system lattice under the strict convention used in this paper that excludes a parent node with a V-structure being the DV. However, we also show that when this constraint is relaxed so that a parent node within a V-structure can be the DV, BN models can offer predictions unique to BN. We also show that (under the above convention) the BN directed system lattice reduces the size of the full BN neutral system lattice by retaining only graphs that give unique predictions of the DV, significantly reducing the search space to find the best BN when prediction of a single DV is the aim. Finally, this paper develops an augmented RA directed system lattice which expands the conventional RA lattice of prediction graphs to include naïve Bayes equivalent graphs. This augmented lattice encompasses graphs in the BN directed system lattice and allows for models of complex systems which are (i) more predictive and/or (ii) simpler and thus both more comprehensible and more generalizable than models restricted to the conventional RA directed system lattice. 

## 2. RA Lattice

### 2.1. RA Neutral Systems

All lattices shown in this paper are for four variables, but the theory and methodology presented in this paper are general and apply to any number of variables. RA neutral systems include only independent variables, i.e., there is no concept in such systems of a dependent variable. A neutral system model thus represents the relationships, graphically and probabilistically, between all the (independent) variables. The graphical representation specifies the independencies among variables. When data are then applied, probabilities represent the strength of the relationships between dependent variables. Neutral system graphs are commonly used in applications where variable clustering is important, such as computer vision and social and biological network analyses. Neutral system analysis is more computationally demanding than directed system analysis, so when one is really interested in predicting specific variables, directed system models are more convenient. 

**Figure 1:**
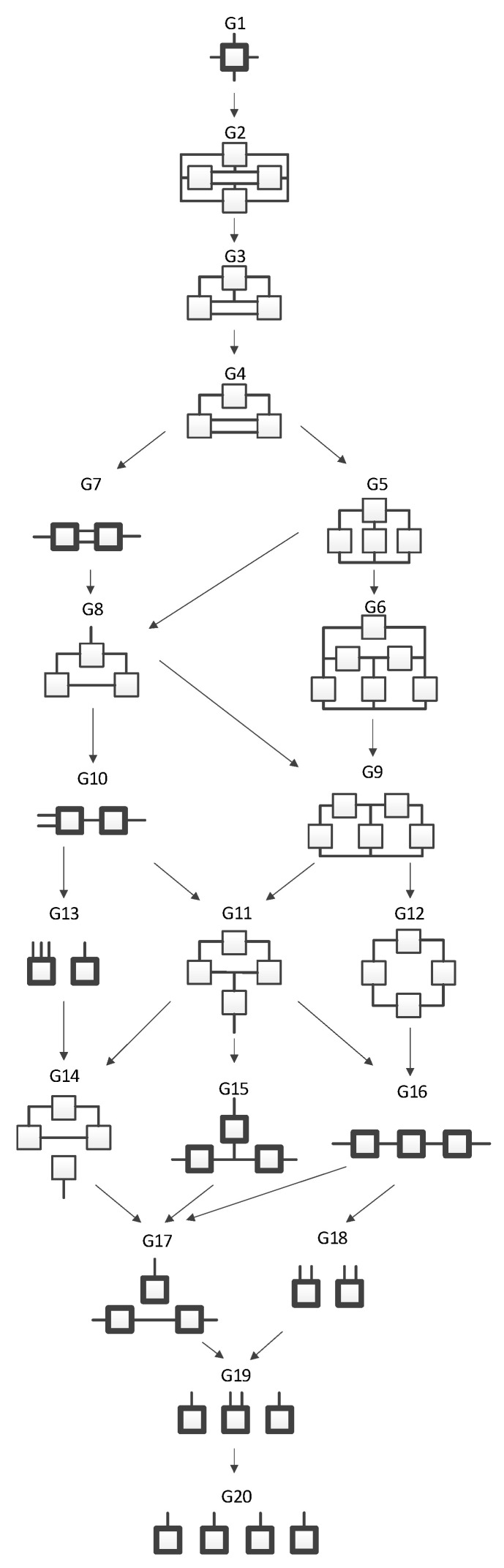
Lattice of four-variable RA neutral system general graphs. Structures with bold boxes (relations) are loopless. All lattices in this paper are for four variables.

The four-variable RA lattice of neutral system general graphs (Figure 1), [7,9], represents all four-variable RA graphs with unique independence structures. Bold graphs do not have loops while non-bold graphs have loops. In these graphs, lines (including branching lines) are variables; boxes are relations. Where only two lines extend from a box, the relation is dyadic. If more than two lines extend from a box, the graph is a hypergraph. Where two or more specific graphs have the same independence structure, regardless of variable labels, they are part of the same general graph equivalence class. For example, the left-most and right-most variables in G7 are independent of one another given the two central variables that connect both relations; this results in the general independence structure (. ⊥.. | …, ….), where each different number of dots indicates a different variable, but does not specify its actual identity. The expression says that the first variable is independent (“⊥” is the symbol used in this paper for independence) of the second variable given (“|” is the symbol used in this paper for “given”) the third and (the comma “,” represents a logical “and”) fourth variables.

G1 is the most complex general graph, in which the variables are connected in a tetradic relation. Graphs below G1 reflect increasingly less complex decompositions of G1, ending with G20 which has complete independence among the variables. Arrows from one general graph to another represent hierarchy such that going from the parent graph (the source of the arrow) to the child graph (the terminus of the arrow) results from deleting one relation from the parent graph.

In this paper, when the variables of a general graph are labeled in RA or BN, it is called a specific graph, which is a unique probabilistic model given the data. For RA, given data and after labeling all the variables, there is only one specific graph for any general graph. By contrast, as explained in the Section 3, (beginning in the Section 3.2.1), two or more topologically different BN general graphs can have the same probability distribution; such equivalent graphs have the same underlying set of independencies even though they are topologically different; they are said to constitute a ‘Markov equivalence class’ [28].

**Figure 2:**
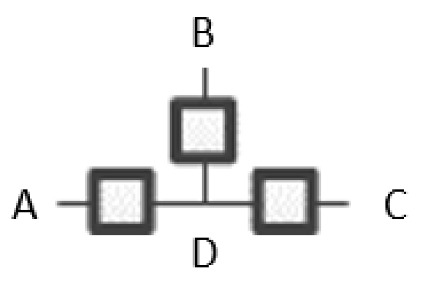
RA specific graph G15, AD:BD:CD.

RA graphs can include pairwise and non-pairwise relations. For example, graph G15 has four lines (variables) and three boxes (relations). One line connects to all three boxes, meaning one variable is included in all three relations, and separately a single line representing one of the other three variables extends from each box. Because only two lines extend from any given box, all relations in G15 are pairwise (dyadic). Figure 2 shows G15 with labels (A, B, C, D) added for the variables, yielding a specific structure having dyadic relations AD, BD, and CD. In RA notation, this graph is AD:BD:CD, the colon represents independence among relations. The notation AD:BD:CD encodes the independencies (A ⊥ B, C | D), (B ⊥ C | D). The example in Figure 2 represents one of four specific graphs for the general graph G15, the other possible permutations are AB:AC:AD, BA:BC:BD, CA:CB:CD. These permutations have the same general independence structure (. ⊥.., … | ….), (… ⊥ … | ….), but given data, produce different conditional probability distributions. 

**Figure 3:**
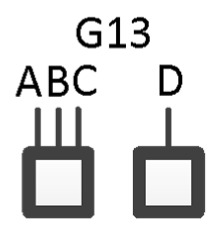
RA specific graph G13.

In contrast to graph G15 which includes dyadic relations only, graph G13 in Figure 1 is a hypergraph, with three lines extending from one box and a single line extending from the other. This could, for example, represent four variables A, B, C and D, where A, B, and C label the three lines extending from one box, and D labels the single line extending from the other box. Figure 3 shows this specific graph, which in RA notation is ABC:D with the independence structure of (D ⊥ A, B, C). This example represents one of four specific graphs for general graph G13, the other three being, ABD:C, ACD:B, and BCD:A with independencies of (C ⊥ A, B, D), (B ⊥ A, C, D), and (A ⊥ B, C, D), respectively. Given data, each of these four specific graphs (ABC:D, ABD:C, ACD:B, and BCD:A) generates a unique probability distribution.

**Figure 4:**
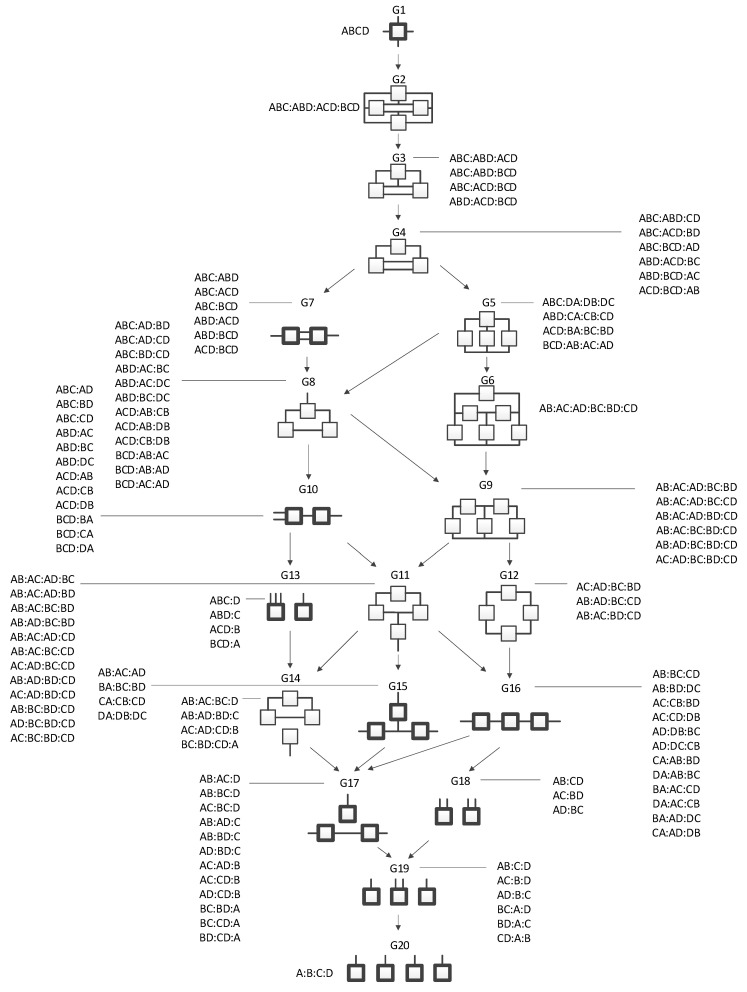
Lattice of RA neutral system general and specific graphs.

Figure 4 shows all of the general graphs from Figure 1 as well as all of the specific graphs associated with each general graph. There are 20 general graphs in the RA lattice and 114 specific graphs. 

#### Searching the RA Neutral System Lattice

The data are the top of the lattice, i.e., G1 ABCD, and one searches the lattice to find a good representation of the data. The lattice can be searched from the top down or from the bottom up or from some other starting model. Typically, a reference model, a specific graph, is selected to begin the search. Commonly, it is the independence (bottom) model, G20 A:B:C:D from Figure 4, that is selected as the reference model, and the lattice is searched upward to find the best model. The lattice may also be searched downward starting from the saturated (top) model, G1, or from a reference model in-between the bottom or top, searching up or down. The starting model does not have to be the reference model, but this is often the case.

Commonly, when the lattice is being searched, the goal is to find a model (a specific graph) that adequately represents but is less complex than the data. This best characterizes a search downwards that (typically) starts from G1. When searching down the lattice, the goal is to search as far down the lattice as possible, resulting in the greatest complexity reduction from the reference model, while incurring the least amount of information loss, so that the model still adequately represents the data. Finding a simpler representation of the data reduces the complexity of the system under observation, allowing for greater understanding of the most important underlying relations. Alternatively, the goal is to find a model, a specific graph, that captures as much of the information in the data as possible, as long as its difference from mutual independence of the variables, i.e., G20 in Figure 4, is defensible, so the model is not overfit, and its application to new data is likely to be more successful. This best characterizes a search upwards that (typically) starts from G20. For directed systems where prediction of a single DV is the aim, a high information model is one that gives maximal reduction of the Shannon entropy (uncertainty) of the DV. 

Given data, specific graphs can be tested for statistical significance. The Chi square statistical test can be used to test the difference between any candidate model and a reference model, usually the data, G1, or the independence model, G20. As an alternative or in addition to such a statistical test, the Bayesian Information Criteria (BIC) and the Akaike Information Criteria (AIC) are among the other measures that can be used to decide on the best model. 

### 2.2. RA Directed Systems

#### 2.2.1. Conventional Directed System Lattice

**Figure 5:**
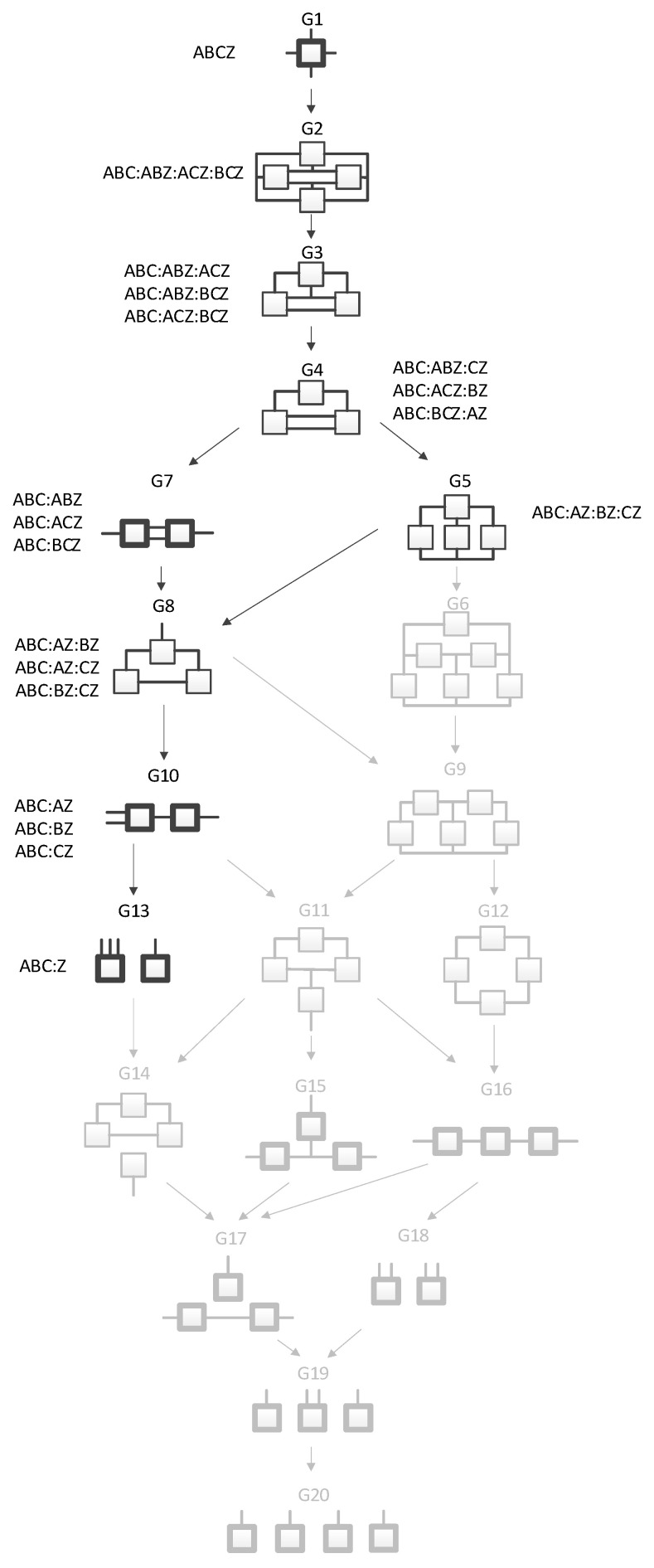
Conventional RA directed system lattice. Structures with boxes (relations) in bold are loopless.

The RA lattice of directed systems shown in Figure 5 is a sub-lattice of the complete neutral system lattice of Figure 1. The purpose of the directed system lattice is to organize models that make an IV-DV (explanatory-response) distinction and where prediction of the DV is the sole aim. In contrast, the neutral system lattice organizes models that do not make any IV-DV distinction; these models do not focus on a single response variable. There are fewer general graphs in the directed system lattice compared to the neutral system lattice because, by convention, we care only about models whose predictions of the DV are different and are not interested in identifying relations among the IVs. The word ‘directed’ in RA ‘directed systems’ has a meaning that is different from the meaning of the same word in BN ‘directed acyclic graphs’. In RA ‘directed systems’, this word means that the focus of modeling is on the relation of the dependent variable to the independent variables. It does not imply directionality of edges from the IVs to the DV as this word means in BN ‘directed acyclic graphs’.

In the neutral system lattice of Figure 1, any of the variables can be part of any relation. In contrast, in the standard directed system lattice, by convention, all of the IVs are always included in one of the relations (the “IV relation”); the other relations in the model include predictive IV-DV interactions (or the DV alone if there are no such interactions). In this paper, the DV in directed system specific graphs is called “Z” and the IVs are called A, B, C, and so on. For example, the first specific graph listed under G3 from Figure 5, ABC:ABZ:ACZ, has all three IVs in the first relation, followed by two IV-DV relations. Aside from allowing for the presence of relations among the IVs (without specifying any such relations), the model says that there is a relation in which A and B might predict Z and another relation in which A and C might predict Z; the net predictive relation between A, B, C and Z is a maximum entropy fusion of these two predictive relations. 

General graph G13 (ABC:Z) from Figure 5 represents independence between the IVs (ABC) and the DV (Z), thus there is no relation between the IVs and the DV, and graph G1 (ABCZ, which is not written as ABC:ABCZ because ABC is embedded in ABCZ) represents complete dependence among the IVs and the DV. It should be noted; however, that the directed system lattice of Figure 5 is not entirely exhaustive. What restricts this lattice is that all models include the “IV relation”; this makes these models hierarchically nested, and amenable to standard statistical tests. There are additional predictive graphs where this restriction is dropped that produce different predictions of Z than the models of Figure 5; these additional graphs are discussed in the following Section 2.2.2. 

Figure 5 shows all directed system general and specific graphs for four variables. The graphs that are greyed represent graphs from the neutral system lattice from Figure 5 that are not part of the directed system lattice because they do not offer unique predictions of the DV. There are nine directed system general graphs and 19 specific graphs in contrast to the neutral system lattice, which has 20 general graphs and 114 specific graphs. 

#### 2.2.2. Augmented Directed System Lattice

**Figure 6:**
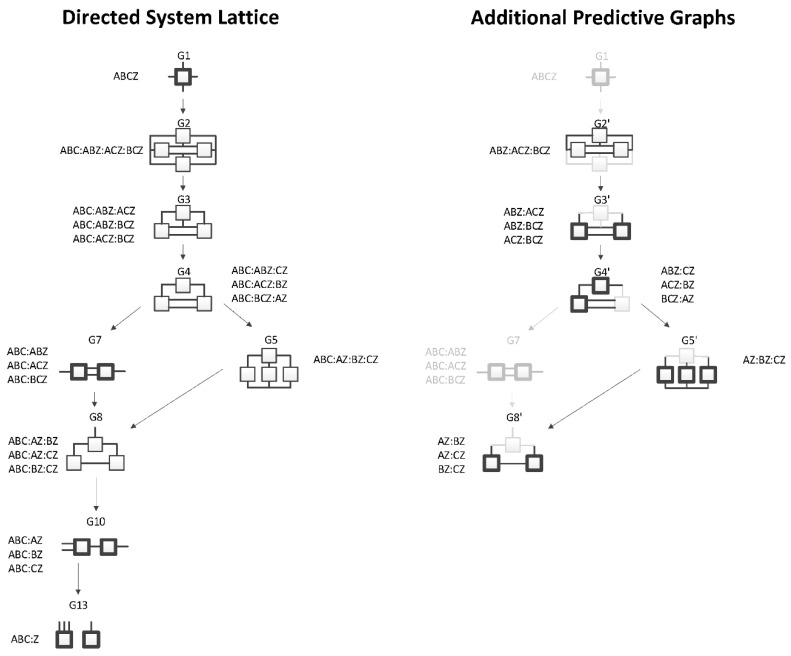
Conventional RA directed system lattice and additional predictive specific graphs. Structures with bold boxes (relations) are loopless.

Figure 6 augments the conventional directed system lattice (on the left) of Figure 5 with a lattice of additional predictive graphs (on the right). These additional graphs offer unique analytical results, but that are not typically included when searching the hierarchically restricted directed system lattice. 

In Figure 6, the graphs in the Additional Predictive Graphs lattice are denoted by an apostrophe to identify that the original graph was altered by removing the IV relation. For example, the bottom relation in graph G2, interpreted as the IV relation, ABC, was removed to produce an additional predictive graph G2′. This general graph has only one specific graph, ABZ:ACZ:BCZ, which is analytically different from G2 (ABC:ABZ:ACZ:BCZ) because the ABC term in graph G2 imposes a constraint among the IVs that is not imposed in graph G2′. Because G2′ does not follow the standard directed system convention of including the IV terms in a first relation, it produces a different prediction of Z. The apostrophe-marked graphs are less complex than the graphs from which they are derived, and so should also be considered in searches for good predictive models. G5′ and G8′ from Figure 6 represent naïve Bayes equivalent RA graphs; G4′ is also a naïve Bayes-like graph. This is discussed in Section 3.3. 

**Figure 7:**
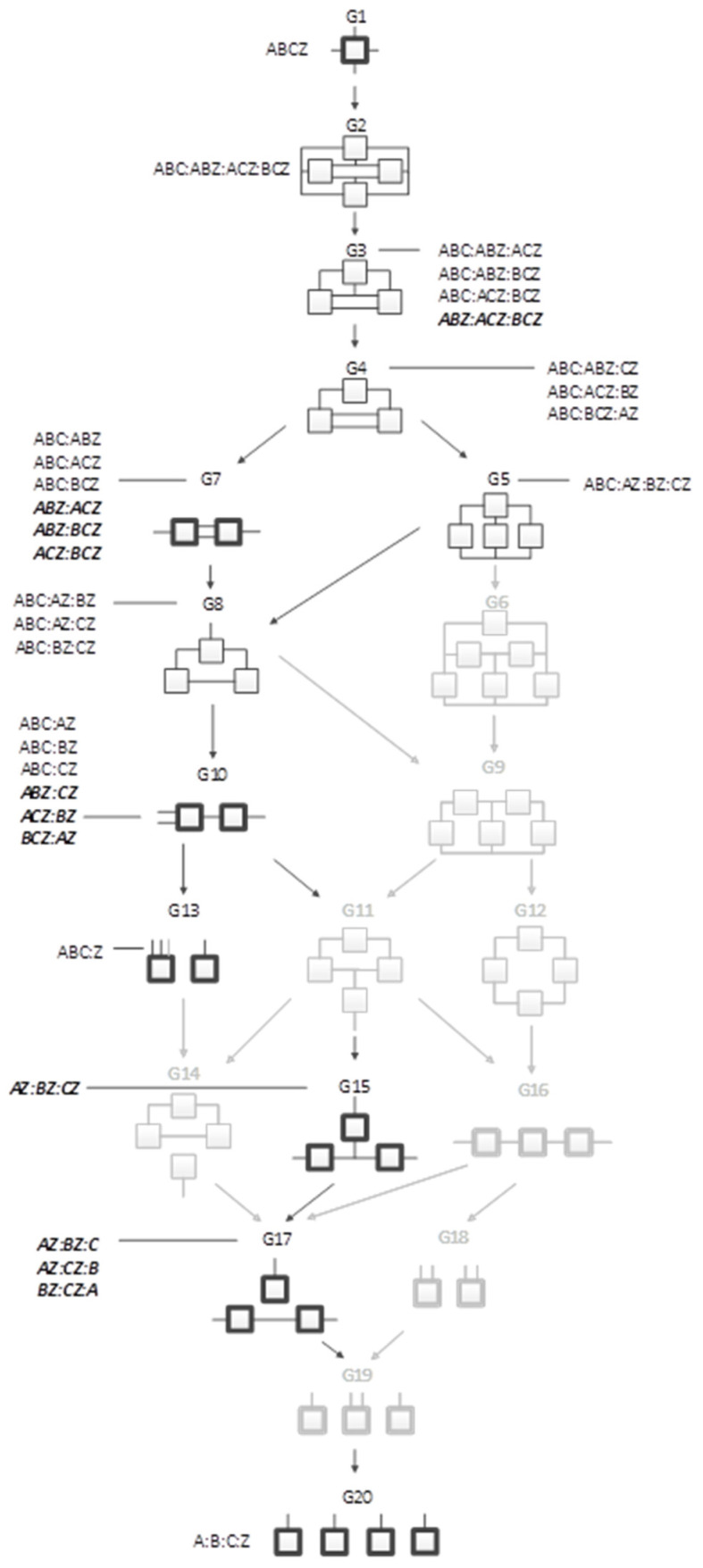
Augmented RA directed system lattice. Structures with bold boxes are loopless; model names in bold are augmentations.

A merger of the conventional directed system lattice with the additional predictive graphs of Figure 6 gives the augmented directed system lattice in Figure 7. The specific graphs from G2′ and G3′ from Figure 6 are members of general graphs G3 and G7, respectively. Three general graphs are added to the augmented lattice, namely G10, G15 and G17; these are G4′, G5′, and G8′ from Figure 6, the naïve Bayes or naïve Bayes-like equivalent RA general graphs. All of the specific structures that are added to the augmented lattice are denoted in bold letters in Figure 7. G13 is the independence model for the conventional directed system graphs. The augmented lattice also includes G20 A:B:C:D, which is the natural independence model for the additional predictive graphs that do not include the IV term (ABC). Including these additional predictive graphs in the directed system lattice increases the number of predictive general graphs from nine in the conventional directed system lattice to 12 in the augmented lattice and 19 specific graphs in the conventional lattice to 31 in the augmented lattice.

## 3. BN Lattice

### 3.1. BN Introduction 

A Bayesian Network model, like an RA model, is a type of probabilistic graphical model. BN modeling originated from path models in the early 1900s [18,19] and was expanded as a field of study in the late 1900s by Pearl [21], Neapolitan [20] and others. 

BNs are directed graphs: nodes represent variables, and edges represent relations. The graph structure or topology (variables, edges, orientations of edges) encodes independencies, and thus also dependencies, among the variables identified in a particular graph. Since BNs are directed graphs, edges typically have arrows or some form of notation representing directionality: A→B means that variable B is dependent upon variable A. (This dependency might be interpreted as a causal influence of A on B, but in this paper, we will not address such causal interpretations of BNs.) A is the ‘parent’ of B, which means that they are dependent. One variable is independent of all other variables given its parents. For example, in the BN A→B→C, variable C is independent of A given B, since B is the parent of C.

A BN graph provides the structure from which a probability expression can be derived that describes the relation between variables. For example, the graph A→B provides the structure identifying the dependence between A and B, and probability values define the nature and strength of the relation between A and B. A unique feature of BNs versus other graphical models is in the independencies that are encoded when two edges converge. For example, in A→B←C the edges converge on variable B. If A and C are not directly connected by an edge, this convergence is called a V-structure [29]. This V-structure is interpreted as yielding the conditional distribution p(B|A,C)p(A)p(C), which encodes dependence among A, B, and C, but marginal independence between A and C. The interpretation is that together, but being independent of one another, A and C influence or cause or allow one to predict B. 

BNs are also acyclic graphs, meaning they have no closed paths following the arrows. For example, graph A→B→C→A is disallowed because it contains a cycle. Because BNs are acyclic, inference on all BN graphs can be performed in closed algebraic form. 

The primary differences between RA and BN are two-fold: (1) BNs are directed and acyclic whereas RA graphs are undirected and can have loops or not have loops and (2) some BN graphs contain converging edges, that is one or more V-structures that encode unique independence relations not found in RA graphs. The absence of a V-structure in a BN graph results in this graph being equivalent to some (loopless) RA graph. The presence of a V-structure results in the graph not having an RA equivalent and thus being unique to BN. This is discussed below in Section 3.2.6, in connection with Table 3. 

### 3.2. BN Neutral Systems 

#### 3.2.1. Lattice of BN General Graphs 

As in RA, there are general BN graphs and specific BN graphs; in the BN literature general graphs are referred to as maximally oriented graphs [30], essential graphs [31], equivalence classes of directed acyclic graphs [32], and partially directed graphs (PDAG) [29]. 

**Figure 8:**
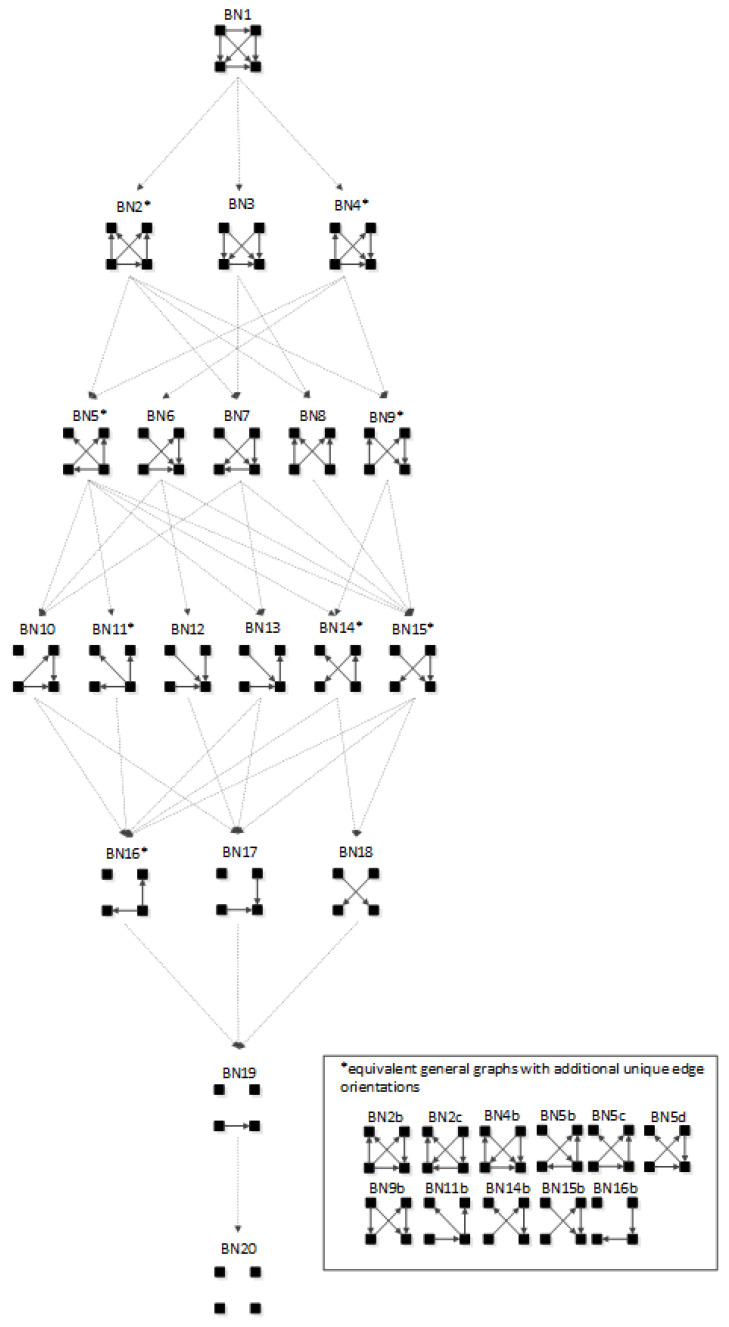
Lattice of BN neutral system general graphs.

In BN general graphs, the graph structure (variables, edges, and orientations of edges) results in a unique independence structure, where specific identities are not assigned to the variables. Figure 8, developed by Harris and Zwick [33], shows all BN general graphs with four variables and their hierarchy. There are 20 BN general graphs in the lattice, i.e., 20 unique independence structures. The procedure to generate this lattice is outlined in Section 3.2.6.

In Figure 8, general graphs are labeled BN1, BN2…BN19, BN20. Solid squares represent variables; edges are represented by directed arrows from one square to another, representing a parent–child dependency relationship. The dashed lines with arrows from one general graph to another represent the hierarchy of general graphs, with parent graphs being above child graphs. Child graphs result from the deletion of one edge from the parent graph. The insert on the bottom right indicates structures that are topologically different from graphs in the lattice marked with asterisks but have identical independence structures to these marked graphs and thus are Markov equivalent (the topological difference cannot be removed by any labeling of the variables). For example, BN2b and BN2c in the insert are topologically different but have the same independence structure as BN2* in the lattice. These additional representations are discussed below in Section 3.2.2.

Table 1 summarizes the RA and BN terminology and supports the discussion of BN that follows. Entries in the table for RA general and specific graphs (the lattices of general and specific graphs from Figure 1 and Figure 4, respectively) have already been discussed above. The discussion that follows this table will explain the additional representations of BN general graphs in the insert of Figure 8, and will derive the lattice of specific BN graphs summarized in Figure 14 presented below in Section 3.2.5. 

#### 3.2.2. Additional Representations of BN General Graphs 

There are 20 general graphs in the BN lattice. However, eight of these, marked with asterisks in Figure 8, namely BN2*, BN4*, BN5*, BN9*, BN11*, BN14*, BN15*, and BN16*, represent Markov equivalence classes that include additional unique edge topologies that have identical probability distributions when applied to data. These additional topologies, shown in the insert at the bottom right of Figure 8, cannot be made equivalent to the representative graphs (those with asterisks) by any 1:1 mapping of unlabeled variables. This property, described by Heckerman [34], who showed that BNs with differing edge topologies can have the same independence structure and thus the same probability distribution, is unique to BN and is not found in RA, where there is a single unique representation of each RA general graph. All general graphs in Figure 8 without an asterisk have no Markov equivalent representations. 

**Figure 9:**
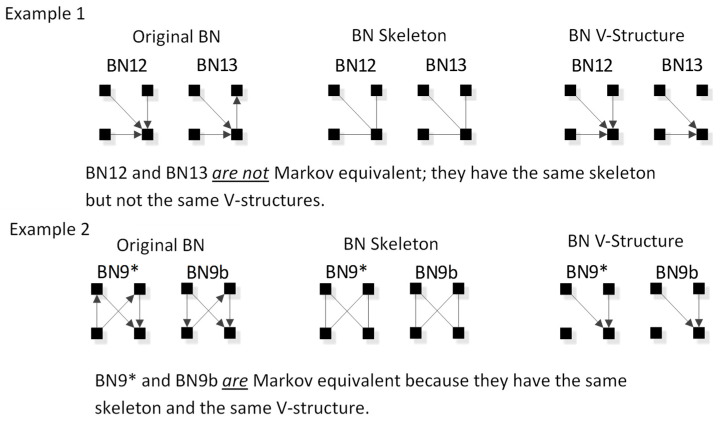
Examples of Markov equivalence tests.

Two Bayesian Networks are Markov equivalent if and only if they have the same skeleton and the same V-structure [28], resulting in the same underlying independence structure. The skeleton of a graph is its undirected representation. As already defined, a V-structure occurs when two or more directed edges that are not themselves directly connected by an edge converge on a single node. Figure 9 shows an example of Markov non-equivalent (Example 1) and equivalent (Example 2) BN general graphs.

**Figure 10:**
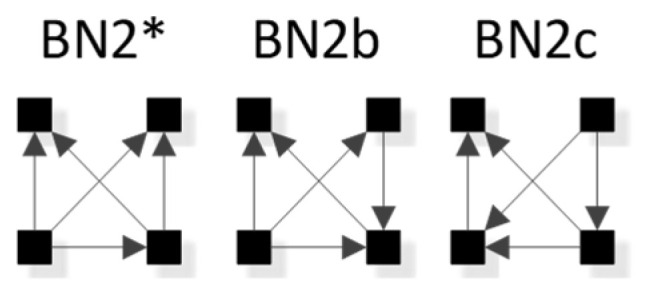
BN2*, BN2b, BN2c.

BNs that are Markov equivalent define an equivalence class; this is illustrated by BN2* in Figure 10 for which two other general graphs (BN2b and BN2c) included in the insert at the bottom of Figure 8 are in the same equivalence class. All three general graphs are Markov equivalent because they have the same skeleton and V-structures, and thus the same independence structure, but they have semantically different edge orientations. BN2* was chosen arbitrarily to represent this equivalence class and its unique independence structure. BN2b and BN2c have the same independence structure, and for corresponding variable labels, have identical probability distributions.

**Figure 11:**
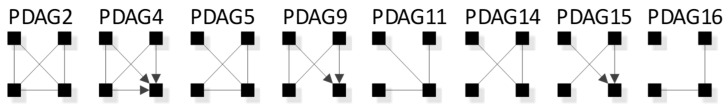
PDAGs for graphs in Figure 8 insert.

A BN general graph is represented in the literature by an unlabeled PDAG [29], also known as a Maximally Oriented Graphs [30], Essential Graph [31] and equivalence classes of directed acyclic graphs [32]. In a PDAG, edges can be directed, undirected or a mix of directed and undirected. A PDAG includes edge direction when a V-structure is present and removes edge direction when no V-structure is present. If there are no V-structures in a given BN, all edges are undirected in its PDAG representation. Figure 11 shows the PDAG representation of the graphs shown in the insert at the bottom of Figure 8. (PDAG2 encompasses BN2b and BN2c, etc.) Undirected edges can have either direction as long as a cycle is not created and also a V-structure is not created that is represented by another BN general graph. For example PDAG16, labeling variables A, B, C, D in order of left to right, top to bottom could be oriented as B←D→C (BN16*) or B→D→C (BN16b) (or its mirror image) but could not be oriented as B→D←C, because that creates a V-structure resulting in a different independence structure represented separately by BN17.

Although representation of an entire Markov equivalence class in a single PDAG is useful, the PDAG does not visibly display the fact that semantically different edge topologies inhere in many BN general graphs (in 8 of 20 general graphs in the four-variable lattice). Use of Figure 8 to display the BN general graph lattice opts instead to show representatives of these classes and also their alternative topologies in the insert at the bottom of the figure.

#### 3.2.3. BN Specific Graph Notation

A BN specific graph is simply a labeled BN general graph. As summarized in Table 1, we use the terminology of “specific graph” for what in the BN literature is called a labeled maximally oriented graph or essential graph or equivalence class of directed acyclic graphs or partially directed graph; these four different terms all refer to the same thing. All specific graphs for a given BN general graph class can be generated by permuting all possible variable labels. Given data, two BN specific graphs with different labels from the same BN general graph class will produce different probability distributions. 

**Figure 12:**
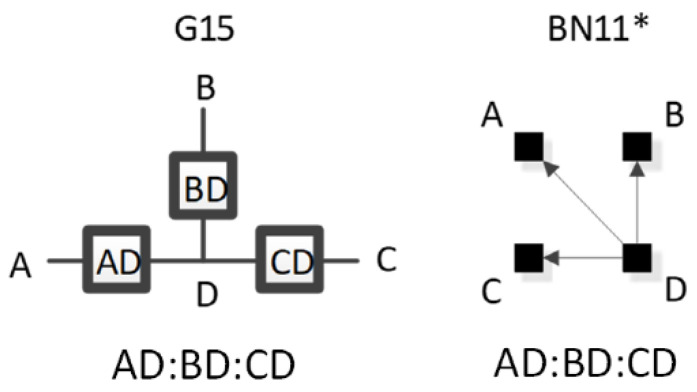
RA and BN notation example, without subscripts.

The notation that we use for BN specific graphs is derived from the RA notation described previously. As in RA, the colon represents marginal or conditional independence among variables and relations. For example, Figure 12 shows a labeled version of RA general graph G15 and BN general graph BN11* which can also equivalently be represented by BN11b, both of which have the same independencies (A ⊥B, C | D), (B ⊥C | D), the same conditional probability distribution p(A|D)p(B|D)p(C|D)p(D) and thus the same notation AD:BD:CD.

**Figure 13:**
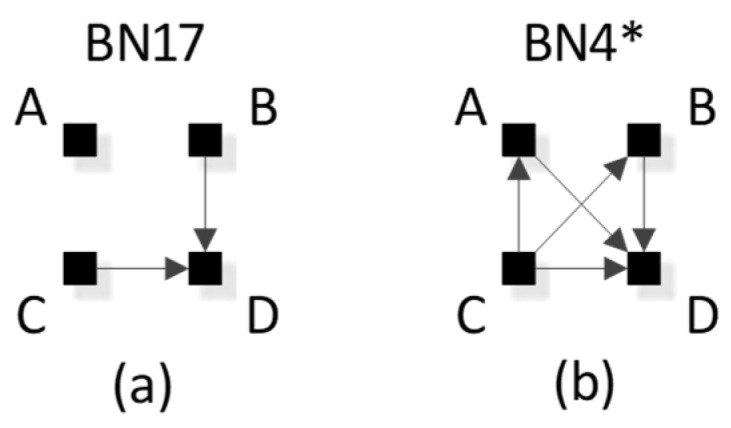
BN notation examples with subscripts. (**a**) BCD_B:C_:A; (**b**) ABCD_AC:BC_.

RA notation must be modified to accommodate the V-structures that are unique to BNs and not found in RA; this is done by adding subscripts that specify the independence relations encoded by the V-structures. (For a BN graph without a V-structure, BN notation is identical to the RA notation.) For example, BN17 in Figure 13a has the notation BCD_B:C_:A, where the colon between BCD_B:C_ and A states the independency (A ⊥ B, C, D), namely that A is marginally independent of B, C, and D. The subscript _B:C_ states marginal independence between B and C within the triadic, dependent, BCD relation. Figure 13b shows the more complex BN4, which has a V-structure in which A, B, and C have arrows going to D; this means that it has a tetradic dependency between A, B, C and D, which will be reflected in a p(D|ABC) in the probability expression for this graph. The graph also has the single independency (A ⊥ B | C). The notation for this graph is thus ABCD_AC:BC_, which preserves the dependency between A, B, C, and D, and also encodes the conditional independence between A and B given C. (In RA, this conditional independence is expressed by saying that T(AC:BC)=TC(A:B)=0, where T is information-theoretic transmission.)

#### 3.2.4. BN Independencies and Probability Distributions

As has been repeatedly stated in the above discussion, the marginal or conditional independence between variables and relations is what uniquely specifies an RA or BN model. “It is known that the statistical meaning of any causal model can be described economically by its stratified protocol, which is a list of independence statements that completely characterize the model” [22,23,28]. The method to determine BN independencies is known as D-separation, and is described in the Appendix A.2. To determine the list of independence statements that completely describe any BN, D-separation is applied to *all possible* independence statements for a given BN. Those satisfying independence among variables are retained and represent the set of independencies that fully describe the structure of relations within a given BN. For four variables, Table 2 provides all possible independence statements. For a given BN, with node labels and directed edges, all independence statements from this table need to be tested. Independence statements that are satisfied are kept, and represent the set of independencies that fully describe that BN. 

D-separation can also be used to test the Markov equivalence of any labeled BNs. If two BNs have the same independencies as revealed by D-separation tests, they are in the same Markov equivalence class and thus the same BN general graph. The prior section, however, provided a simpler way, illustrated above in Figure 9, to test for Markov equivalence of two BNs with different edge topologies.

#### 3.2.5. Lattice of BN General and Specific Graphs

The BN literature on lattices predominately focuses on search algorithms to find the best BN given a scoring metric. Implicit in these search algorithms is a lattice of candidate graphs being explored in search of the best model. Chickering [35] and others have shown the search problem to be NP-hard, with four variables there are 543 possible BNs, with 10 variables there are O(10^18) [36]. Because of this, research in this area has focused less on characterizing exhaustively the lattice of BN graphs, and more on advancing search heuristics to efficiently traverse the lattice to identify the best BN given a scoring metric [37,38,39,40,41,42,43,44,45,46], and others.

Heckerman [34] first showed that BNs with differing edge topologies can have the same independence structure and the same probability distribution, herein described as BN specific graphs. In contrast to heuristics that search all BNs, search heuristics for BN specific graphs have proven to be more efficient because they reduce the dimensionality of search space [29,31,32,40,47,48,49,50], and others. For four variables, this approach reduces the search space from 543 BNs to 185 BN specific graphs [31]. These 185 BN specific graphs can be summarized by 20 BN general graphs all with unique independence structures when variable labels are removed. 

**Figure 14:**
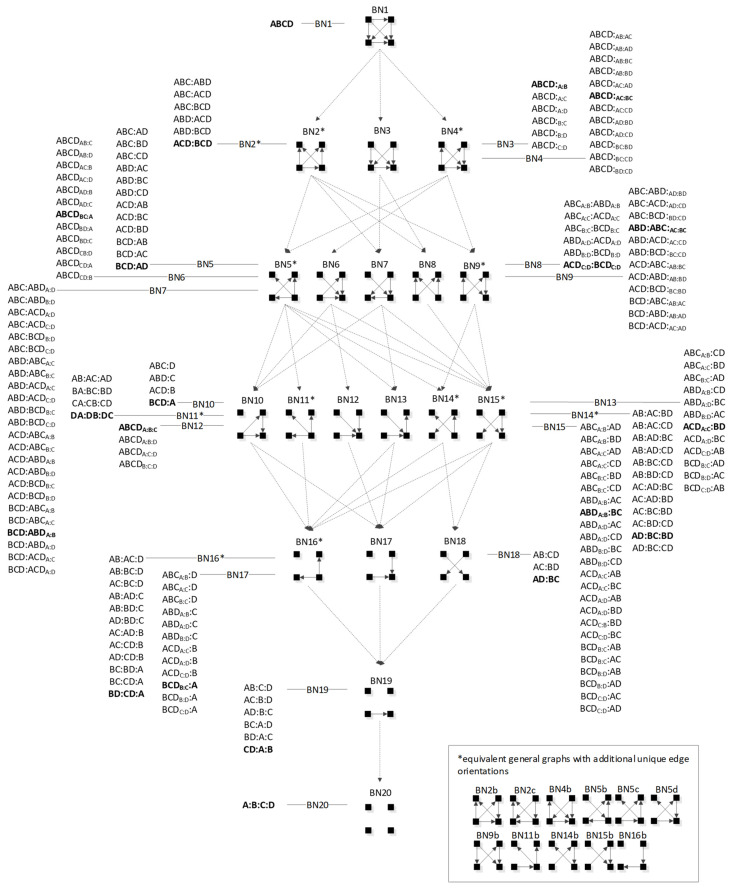
Lattice of general and specific BN neutral system graphs.

Building from the RA work of Klir [8] and Zwick [14], and the BN work of Pearl [21,22,23,51], Verma [28], Heckerman [34], Chickering [29,35,40,52,53], Andersson [31], Rubin [54], and others, the following procedure was used to generate the four variable BN general and specific graph lattice of Figure 14 in a way that can be integrated with the RA general graph lattice. While this procedure is applied in this paper to four variables, it could in principle be used for any number of variables, although of course as the number of variables increases the effort required increases exponentially.

#### 3.2.6. BN Neutral System General and Specific Graph Procedure

The procedure to generate the BN neutral system general and specific graph lattice for any number of variables is as follows:

1. Assign labels arbitrarily to the *n* solid squares representing variables.
2. Generate all graphs for these *n* variables by permuting all possible edge connections and edge orientations. Eliminate graphs with cycles. The result is the set of all labeled directed acyclic graphs for *n* variables.
3. For each directed acyclic graph, determine its independence structure using the D-separation procedure [55] detailed in Appendix A.2. This identifies which of the independence statements in Table 2 apply to the graph.
4. Collect together all graphs with the same unlabeled independencies. The set of these DAGs comprise a general graph equivalence class.
5. For each general graph equivalence class, collect together all graphs with the same labeled independencies into specific graph equivalence classes. List the RA notation for each of these specific graphs.
6. Select one specific graph equivalence class to represent the general graph, and from this specific graph equivalence class, select a single edge topology to represent the general graph. List any additional equivalent general graphs with unique edge topologies separately, as was done in the insert in Figure 8 and Figure 14.
7. Organize general graphs into levels based upon the number of edges in each general graph and link hierarchically nested general graphs in the lattice to reflect parent-child general graphs.

Figure 14 shows the result of following this procedure for four variables. This BN general and specific graph lattice can be directly compared with the RA general and specific graph lattice. The RA lattice can also be extended to include the BN lattice. The comparison and extension will be discussed in Section 4.

Table 3 lists specific graph representatives for each of the general graphs in Figure 14. These specific graphs, highlighted in bold in Figure 14, assume that nodes are labeled in the order A, B, C, D from left to right, top to bottom, which is the labeling convention throughout this paper. The notation for a BN specific graph without a V-structure is identical to the RA notation. As in RA, the colon represents marginal or conditional independence among variables. For a BN graph with a V-structure, the notation adds subscripts to represent the independence relations encoded by the V-structure, which are unique to BNs and not found in RA. (See the Section 3.2.3. for more details on this notation). Thus, graphs in Table 3 without subscripts are equivalent to an RA graph and graphs with subscripts are unique to BN. Equivalence and non-equivalence between RA and BN graphs will be discussed in Section 4.

**Figure 15:**
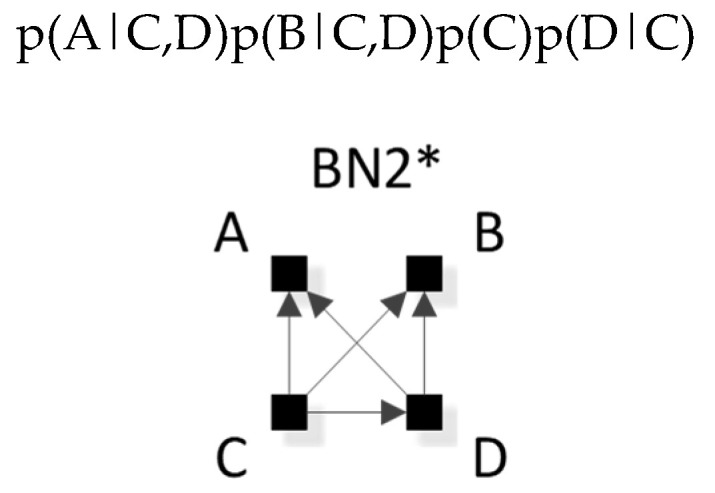
Probability distribution for BN2* example.

Table 3 shows for each BN general graph from Figure 14 a specific graph with its RA notation, probability distribution, and minimal list of independencies resulting from the D-separation procedure. The probability distribution is obtained as follows: (1) For each labeled node of a BN specific graph, list each node’s individual probability expression as the probability of the node given its parents, i.e., p(node | parents); if there are no parents, simply the p(node). (2) Join the list of probability expressions. For example, for BN2* in Figure 15, the individual probability expressions are p(A|C,D) for A, p(B|C,D) for B, p(C) for C, and p(D|C) for D. Joining these gives p(A|C,D)p(B|C,D)p(C)p(D|C). (The table omits the commas for variables that are given in conditional probability terms.)

The equivalence or non-equivalence of RA and BN graphs is discussed in detail in Section 4, below, but Table 3 provides an advanced look at this issue. Any BN general graph with a specific graph example whose RA notation does not include subscripts is equivalent to some general RA graph; there are 10 of these BN general graphs. Any BN general graph with a specific graph example whose notation includes subscripts is not equivalent to any general RA graph; there are also 10 of these BN general graphs, which all have V-structures. 

### 3.3. BN Directed Systems

**Figure 16:**
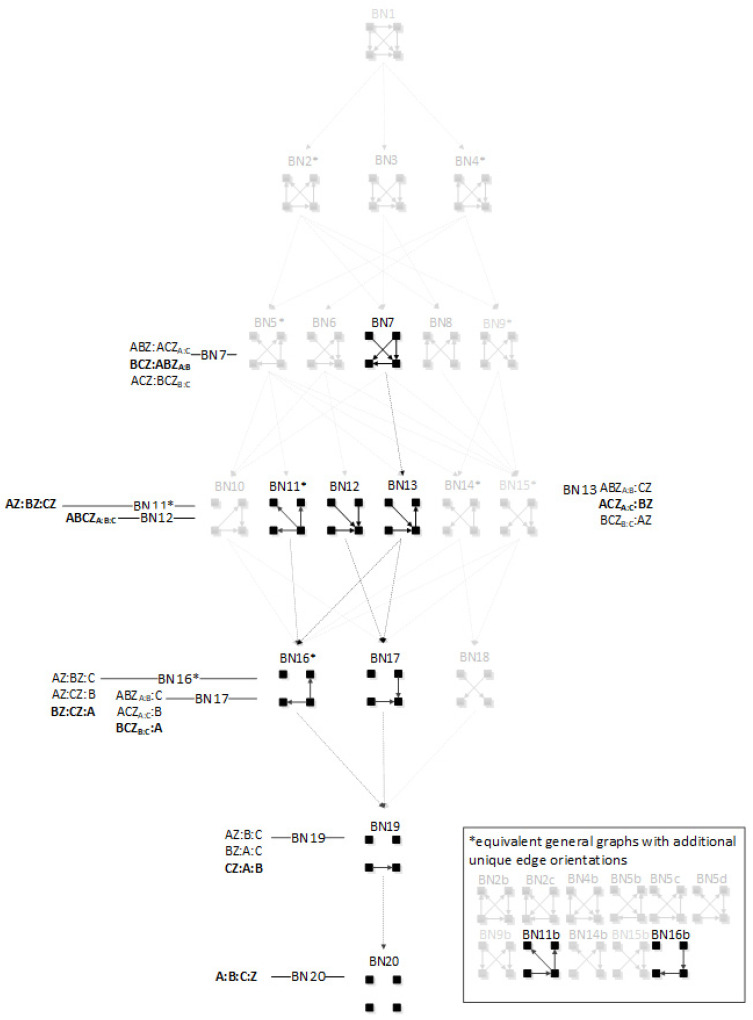
BN directed system lattice.

The BN discussion so far has focused on BN neutral systems in which an IV-DV distinction is not made. This section narrows the focus to BN predictive graphs, analogous to RA directed systems, where the aim is to predict a single DV given the IVs. As in RA, we define Z as the dependent variable in the BN directed system lattice, replacing variable D in the neutral system lattice. We designate as the DV in a given BN any node with the exception of a parent node within a V-structure. That is, we do not consider here the possibility that a parent node within a V-structure could be designated as a DV; this will be discussed further in Section 5. As is the case for RA, many graphs in the neutral system lattice are redundant when the aim is only to predict the DV. The BN directed system lattice of Figure 16, where only graphs with unique predictions of Z are highlighted, is thus a subset of the BN neutral system lattice of Figure 14. For each general graph in Figure 16 with a unique prediction, associated specific graphs are listed. Specific graphs that are bolded correspond to the displayed BN edge orientation and edge connections assuming labeling of nodes from top left, top right, bottom left, bottom right as A, B, C, Z respectively. These bolded specific graphs also correspond to the examples in below Table 4. Graphs not highlighted in Figure 16 are equivalent in their predictions to highlighted graphs. (Asterisks in this figure have the same meaning they have in BN Figure 8 and Figure 14). For two graphs with identical predictions, the graph with the least degrees of freedom was selected. There are eight general graphs and 18 specific graphs in the BN directed system lattice; this is a significant compression of the BN neutral system lattice that includes 20 general graphs and 185 specific graphs. 

Table 4 lists all BN directed system general graphs. When BN graphs are greyed in column 1 it means the graph is equivalent in terms of prediction to a simpler (fewer degrees of freedom) general graph. Column 2 identifies which simpler graph it is equivalent to. General graphs with a blank row in column 2 have no simpler equivalently predicting graph, and are included in the directed system lattice of Figure 16. Column 3 provides specific graph examples of these general graphs and column 4 shows the specific graph probability distributions. Within column 4, only the expressions that are used to predict the dependent variable are highlighted in black. All other non-predictive relations are greyed. For example, BN1, BN3, BN4, and BN6 and BN12 all predict Z in the same way, i.e., p(Z|ABC), thus they are all equivalent in terms of prediction. However, BN12 has the least degrees of freedom and is therefore selected to represent all five of these equivalent general graphs.

## 4. Joint RA-BN Neutral System Lattice

### 4.1. Joint RA-BN Neutral System Lattice Introduction

**Figure 17:**
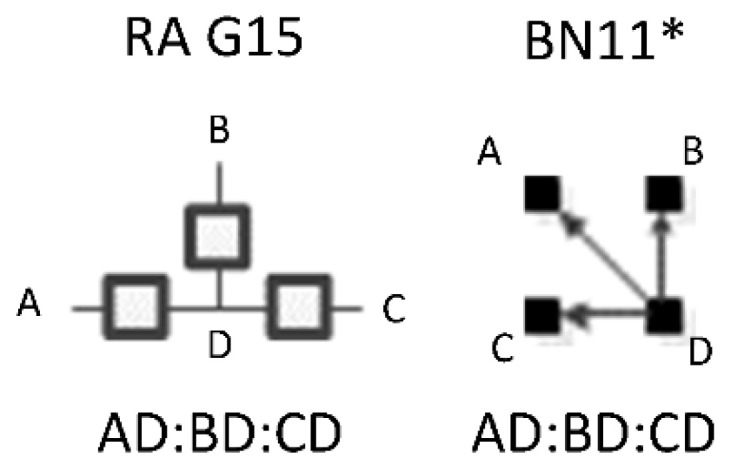
G15 and BN11* specific graph example.

This section integrates the RA and BN neutral system general graph lattices using the four variable Rho lattice [7]. Combining the Rho, RA and BN lattice creates a larger and more descriptive lattice than any previously identified in the literature. The lattice identifies independence structures unique to RA or to BNs, and independence structures that are equivalent across RA and BN. Equivalence is in terms of independence structure as described separately for RA in the Section 2, and BN in the Section 3. Where two or more graphs, RA or BN, have the same general independence structure regardless of variable labels, they are equivalent. General independence structure is represented with independence statements without labels. For example, (. ⊥ .. | …), one variable is independent of another, given a third. Consider, for example, RA general graph G15 and BN general graph BN11 have the same general independence structure (. ⊥ .., … | …), (.. ⊥… | …), thus they are equivalent. Two specific graphs are equivalent if they have the same independence structure given variable labels. For example, using RA general graph G15 and BN general graph BN11 again, Figure 17 shows these general graphs with variable labels added making them specific graphs. Given these labels, they have equivalent general and specific independence structure, (. ⊥ .., … | ….), (.. ⊥… | …) and (A ⊥B, C | D), (B ⊥ C | D) respectively.

### 4.2. RA-BN Rho Neutral System Graphs

**Figure 18:**
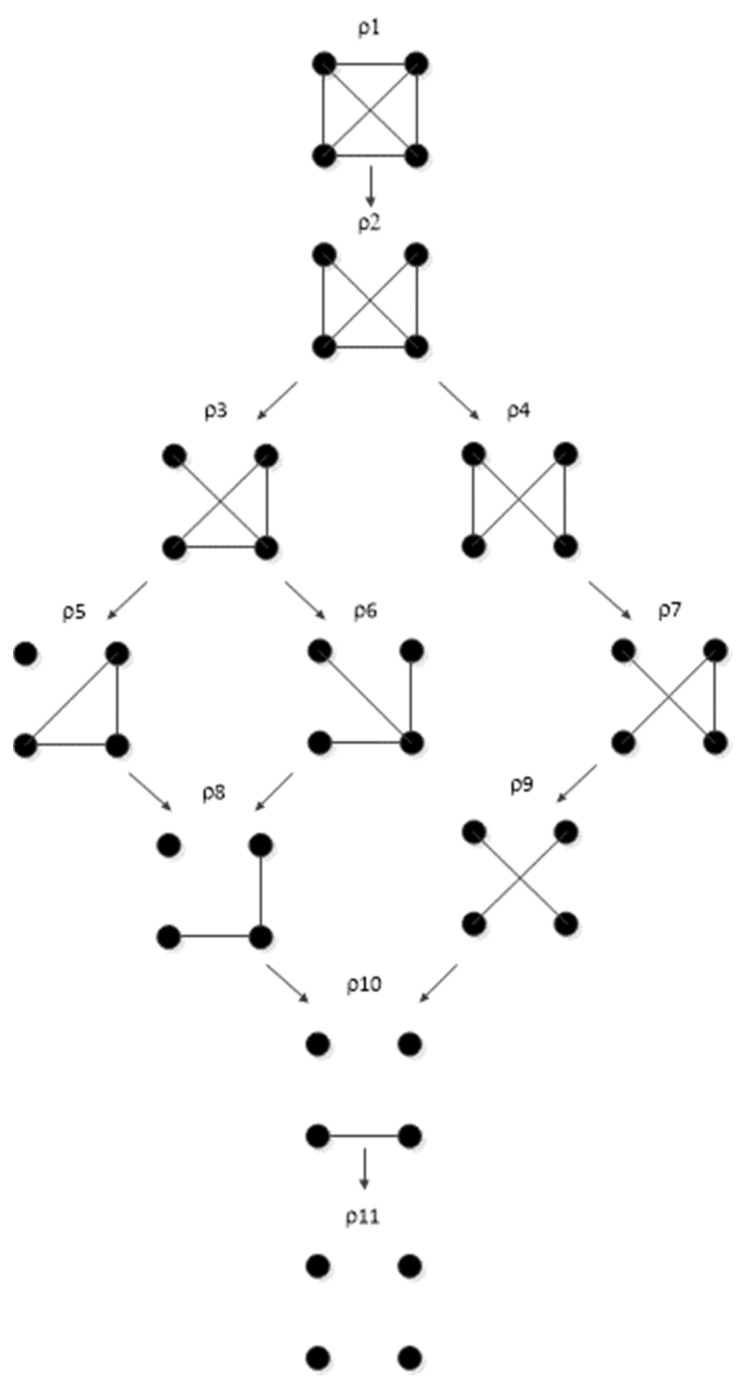
Lattice of four-variable Rho graphs.

The Rho (ρ) lattice of Figure 18 (adapted from Klir [7] (p. 237)) is a simplification of the RA lattice of general graphs and is used here to integrate the RA neutral system lattice with the BN neutral system lattice. The Rho lattice is an even more general lattice than the RA general graph lattice and can map both RA and BN general graphs to one of its eleven structures. A solid dot represents a variable; a line connects variables in the Rho lattice if these two variables are directly connected by any box (relation) in the RA general graph lattice. Arrows from one Rho graph to another represent hierarchy, i.e., the generation of a child graph from a parent graph. ρ1 represents maximal connectedness, or dependence, between variables, and ρ11 represents independence among all variables. Graphs in-between ρ1 and ρ11 represent a mix of dependence and independence among variables. Each RA or BN general or specific graph corresponds to one, and only one, of the eleven Rho graphs. 

### 4.3. Rho and Equivalent RA and BN General Graphs 

Out of 20 RA neutral system general graphs and 20 BN neutral system general graphs, there are 10 RA general graphs, comprising all of the graphs with no loops in the RA lattice that are equivalent to BN general graphs. Each of these RA-BN equivalent pairs corresponds to one of the 11 Rho graphs from Figure 18, with the exception of ρ4. ρ4 has corresponding RA and BN general graphs, but these do not have equivalent independence structures, and are discussed in the following Section 4.3.

**Figure 19:**
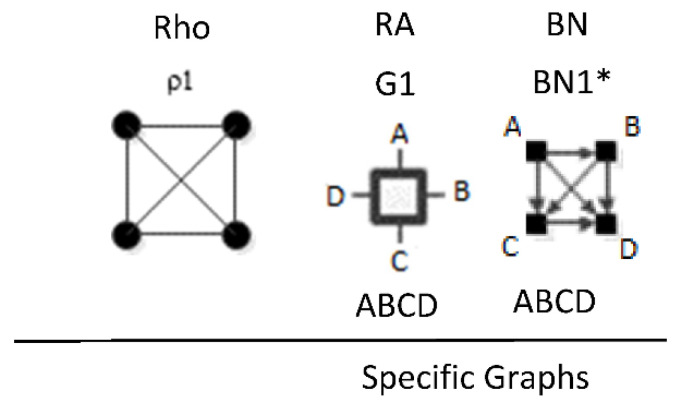
Rho1, G1 and BN1 specific graph.

ρ1 reflects maximal connectedness among all four variables. For both the RA general graph G1 and the BN general graph BN1 from Figure 1 and Figure 8 respectively, there are no independencies among the variables and thus the graphs are equivalent. Both graphs have only one specific graph, ABCD. This is summarized in Figure 19. 

**Figure 20:**
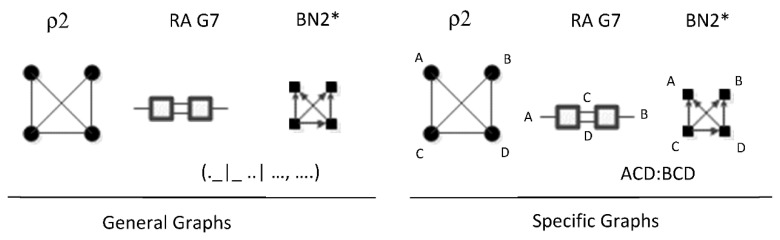
Rho2, G7, and BN2* example.

ρ2 corresponds to RA general graph G7 and BN general graph BN2*, as shown in Figure 20. It is clear how BN2* corresponds to Rho graph ρ2 because visually they are represented in the same way with the exception that the Rho graph has undirected edges. There are two additional BN general graphs (BN3 and BN4*) that correspond to ρ2; however they have no equivalent RA general graph, so they are discussed in the next section which concerns non-equivalent RA and BN general graphs. ρ2, G7, and BN2* represent two three-variable relations with conditional independence between two variables, with general independence structure (. ⊥ .. | …,….). Assigning labels to variables makes it easier to interpret the RA association with ρ2. Figure 20 shows an example with variable labels (one of six possible permutations of variable labels) assigned to RA graph G7 which results in RA specific graph ACD:BCD, in which A is independent of B given C and D, (A ⊥ B|C, D). Assigning labels to the BN graph in Figure 20 yields the same specific graph. Other label permutations yield five other equivalent RA and BN specific graphs: ABC:ABD, ABC:ACD, ABC:BCD, ABD:ACD, ABD:BCD.

**Figure 21:**
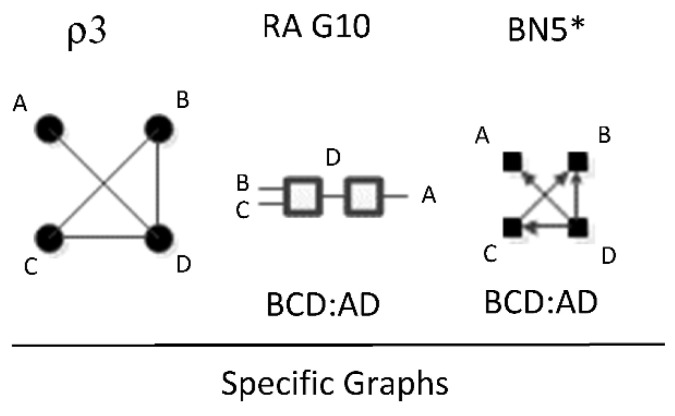
Rho3, G10, and BN5* specific graph example.

ρ3 represents RA graph G10 and BN graph BN5* which have the same independence structure, (. ⊥ .., … |….). Figure 21 shows an example of one of eight RA G10 and BN5* specific graphs, BCD:AD, with independencies (A ⊥ B, C |D). The full list of eight specific RA G10 and BN5* specific graphs are: ABC:AD, ABC:BD, ABC:CD, ABD:AC, ABD:BC, ABD:DC, ACD:AB, ACD:CB, ACD:DB, BCD:BA, BCD:CA, and BCD:DA. G10 has been previously characterized as naïve BN-like.

ρ4 is discussed later in the section on non-equivalent RA and BN general graphs.

**Figure 22:**
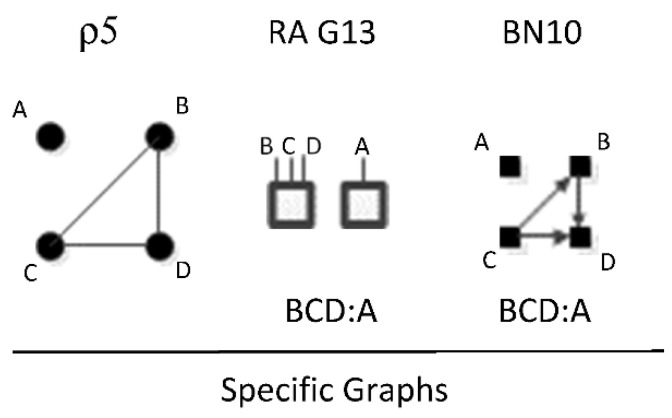
Rho5, G13, and BN10* specific graph example.

ρ5 represents RA graph G13 and BN graph BN10 which have the same independence structure, (. ⊥ .., …, ….) in that they have no independencies in the triadic relation and the fourth variable is independent of all three variables in the triadic relation. Figure 22 shows an example of one of four RA G13 and BN10 specific graphs, BCD:A, with independencies (A ⊥ B, C, D). The full list of RA G13 and BN10 specific graphs are: ABC:D, ABD:C, ACD:B, and BCD:A. 

ρ6 represents RA general graph G15 and BN graph BN11* which have the same independence structure, (. ⊥ .., … | ….), ( .. ⊥ … | ….). There are three dyadic relations in these graphs with one variable present in all three dyadic relations and the other three variables present in only one of three dyadic relations. 

**Figure 23:**
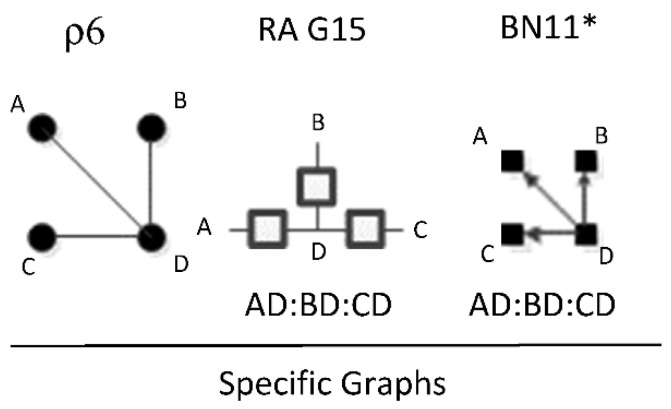
Rho 6, G15 and BN11* specific graph example.

This graph is described in the literature (Zhang 2004) as a naïve BN, simple Bayes, or independence Bayes, because of its simple dyadic relations among variables. What is also clear is RA general graph G15 represents a naïve BN because of its equivalent independence structure. Figure 23 shows an example of one of RA G15 and BN11* specific graphs, AD:BD:CD, with independencies (A ⊥ B, C | D), (B ⊥C | D), and conditional probability distribution p(A|D) p(B|D)p(C|D)p(D).The full list of specific RA G15 and BN11* specific graphs are: AB:AC:AD, AB:BC:BD, AC:BC:CD, and AD:BD:CD.

**Figure 24:**
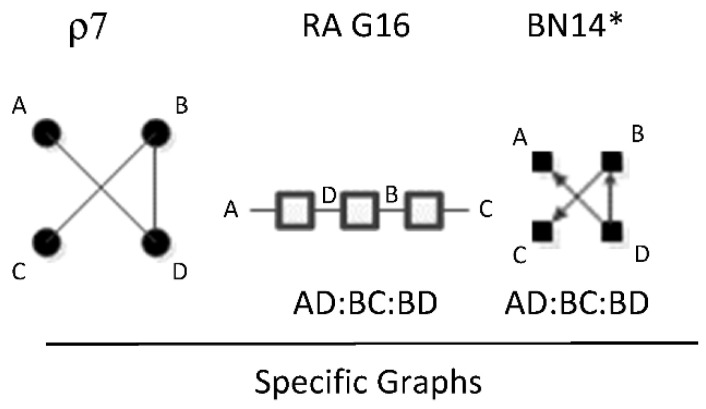
Rho7, G16 and BN14* specific graph example.

ρ7 represents RA graph G16 and BN graph BN14* which have the same independence structure, (. ⊥ .. | ….), (… ⊥ ., …. | ..). Figure 24 shows an example of one of twelve RA G16 and BN14* specific graphs, AD:BC:BD, with independencies (A ⊥ B | D), (C ⊥ A, D | B) and conditional probability distribution p(A|D)p(B|D)p(C|B)p(D). The full list of specific RA G16 and BN14* specific graphs are: AB:AC:BD, AB:AC:CD, AB:AD:BC, AB:AD:CD, AB:BC:CD, AB:BD:CD, AC:AD:BC, AC:AD:BD, AC:BC:BD, AC:BD:CD, AD:BC:BD, and AD:BC:CD.

**Figure 25:**
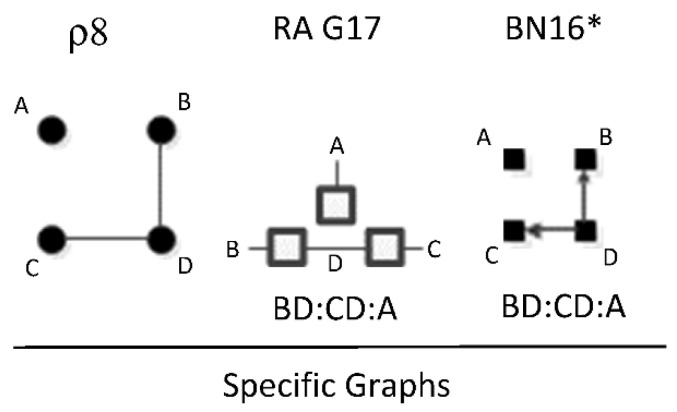
Rho 8, G17 and BN16* specific graph example.

ρ8 represents RA general graph G17 and BN general graph BN16* which have the same independence structure, (.. ⊥ … | ….), (. ⊥ .., …, ….). There are two dyadic relations in these graphs with one variable present in both dyadic relations, and the fourth variable not present in either dyadic relation, and thus independent of the three other variables. This graph is also representative of a naïve BN. Figure 25 shows an example of one of twelve RA G17 and BN16* specific graphs, BD:CD:A, with independencies (B⊥ C | D), (A ⊥ B, C, D), and conditional probability distribution p(B|D) p(C|D)p(D). The full list of specific RA G17 and BN16* specific graphs are: AB:AC:D, AB:BC:D, AC:BC:D, AB:AD:C, AB:BD:C, AD:BD:C, AC:AD:B, AC:CD:B, AD:CD:B, BC:BD:A, BC:CD:A, and BD:CD:A.

**Figure 26:**
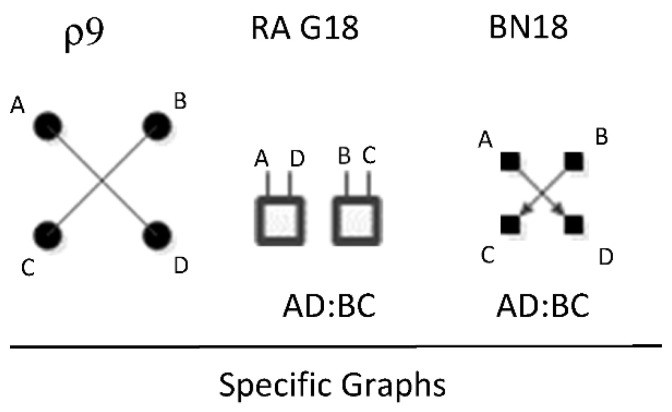
Rho 9, G18 and BN18* specific graph example.

ρ9 represents RA general graph G18 and BN general graph BN18 which have the same independence structure, (. , …. ⊥ .., …). There are two dyadic relations in these graphs with two variables included in one dyadic relation and the other two included in the other. Figure 26 shows an example of one of three RA G18 and BN18 specific graphs, AD:BC, with independencies (A, D ⊥ B, C), and conditional probability distribution p(C|B)p(B)p(D|A)p(A). The full list of specific RA G18 and BN18 specific graphs are: AB:CD, AC:BD, and AD:BC.

**Figure 27:**
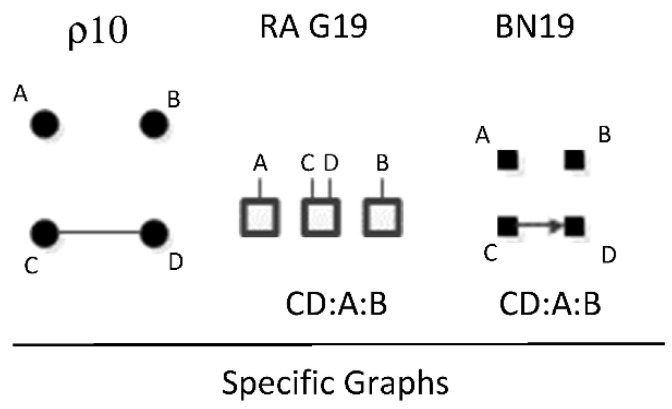
Rho, 10, G19 and BN19 specific graph example.

ρ10 represents RA graph G19 and BN graph BN19 have the same independence structure, (.. ⊥ …, ….), (. ⊥ .., …, ….). There is one dyadic relation and two variables independent of all other variables. Figure 27 shows an example of one of six RA G19 and BN19 specific graphs, CD:A:B, with independencies (B ⊥ C, D), (A ⊥ B, C, D) and conditional probability distribution p(D|C)p(C)p(A)p(B). The full list of specific RA G19 and BN19 specific graphs are: AB:C:D, AC:B:D, AD:B:C, BC:A:D, BD:A:C, and CD:A:B.

**Figure 28:**
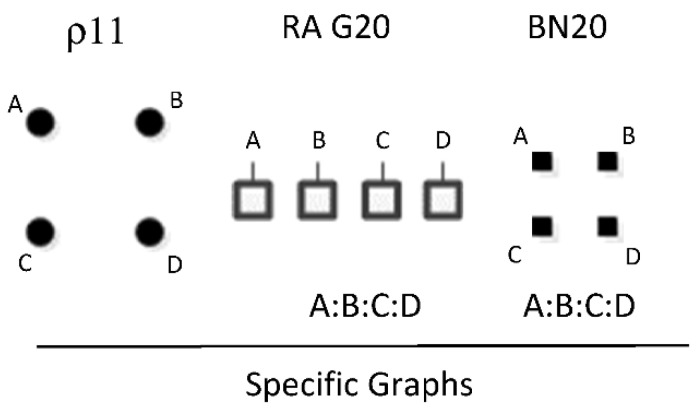
Rho 11, G20 and BN20 specific graph.

ρ11 represents RA graph G20 and BN graph BN20 which have the same independence structure (. ⊥.., …, ….), (..⊥ …, ….), (…⊥....) in which all variables are independent of one another, (A ⊥ B, C, D), (B ⊥ C, D), (C ⊥ D). Figure 28 shows the only specific graph for RA G20 and BN20, A:B:C:D. 

Table 5 summarizes all equivalent RA and BN general graphs, with their associated Rho graph, an example of their specific graph notations and their independences. These specific graph examples align with the BN general graphs of Figure 8 assuming labeling of nodes A, B, C, D in the order of top left, top right, bottom left, bottom right.

### 4.4. Rho and Non-Equivalent RA and BN General Graphs

In addition to the 10 equivalent RA and BN general graphs, there are 10 general graphs unique to the RA lattice and 10 general graphs unique to the BN lattice. All 10 non-equivalent RA general graphs in the four variable lattice have loops and require iteration to generate their probability distributions. BNs are acyclic and have analytic solutions, so there are no BN general graphs that are equivalent to the RA graphs with loops. Since RA graphs are undirected, one might think that there could be some equivalent acyclic directed BN graphs, but this is not the case, because BN graphs that are acyclic when directions are considered but cyclic if directions are ignored have V-structure interpretations, as described previously. All 10 non-equivalent BN general graphs have such V-structures, which encode independence relations unique to BNs. To illustrate: the structure A→B, B→C, C→D, D→A is cyclic and not a legitimate BN structure, but the directed structure of A→B, B→C, C→D, A→D (BN9b from Figure 8), which has the same undirected links, is not cyclic, and is a legitimate BN structure. However, this latter structure is not interpreted as a set of dyadic relations, which would be written in RA notation as AB:BC:CD:AD and contains a loop (RA general graph G12 from Figure 1). Rather, the V-structure consisting of C→D and A→D is interpreted as a triadic relation, which contributes a p(D|AC) to the probability expression, p(A)p(B|A)p(C|B) p(D|AC), which does not correspond to any RA structure. 

### 4.5. Lattice of Rho, RA, BN Neutral System General Graphs

**Figure 29:**
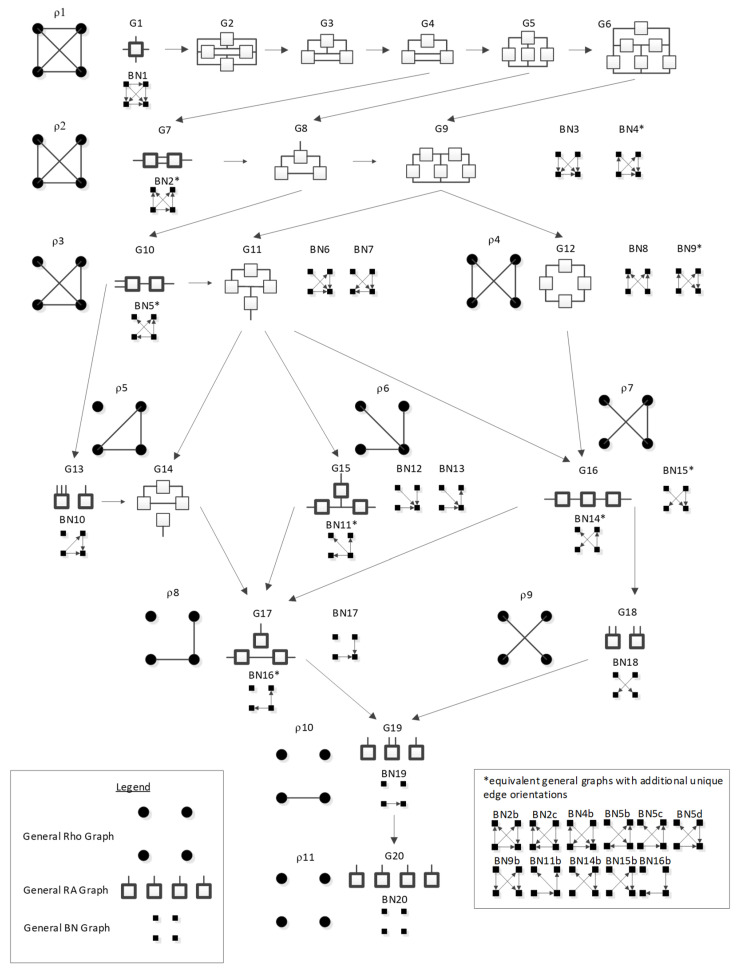
Lattice of 4-variable general Rho, RA and BN neutral system graphs.

The lattice of Rho, RA and BN equivalent and non-equivalent general graphs in Figure 29 was developed from the RA lattice in Figure 1 and the BN lattice in Figure 8. This lattice includes all 10 unique RA general graphs, 10 unique BN general graphs, and 10 RA and BN equivalent general graphs, for a total of 30 unique general graphs. The lattice is organized using the Rho lattice [7]. All 20 RA general graphs and all 20 BN general graphs for each Rho graph are represented in the joint lattice. Within each Rho graph, where RA and BN graphs are equivalent, that is, when their independence structures are identical, the BN graph is placed under the RA equivalent graph. Where RA or BN graphs are not equivalent, representing an independence structure unique to RA or BN, they stand alone.

**Figure A6:**
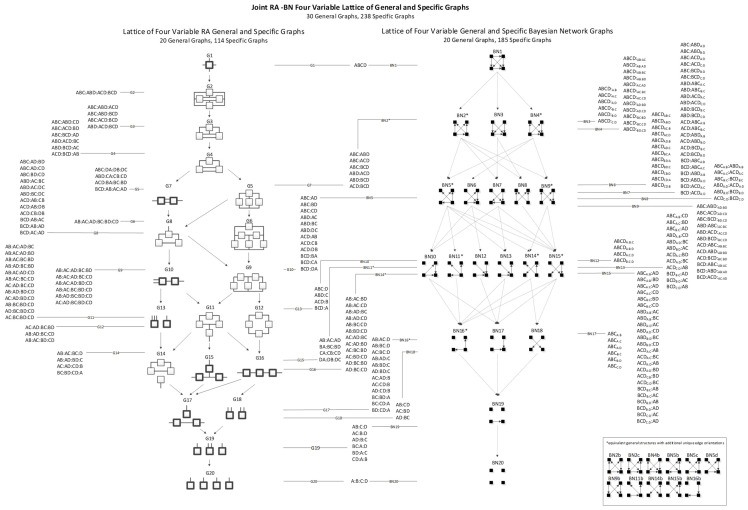
Joint RA-BN lattice of 4 variable general and specific graphs.

Arrows from one graph to another in the joint lattice represent the hierarchy of the RA lattice only. As can be seen in Section 3, the hierarchy of the BN lattice has many more links from parent to child graphs and thus is not a useful representation in the joint lattice. Additionally, Figure A6 in Appendix A includes the Joint RA-BN lattice of general and specific graphs. This lattice shows 53 unique RA specific graphs, 124 unique BN specific graphs, and 61 RA-BN equivalent specific graphs, for a total of 238 combined, unique, RA and BN specific graphs.

### 4.6. Joint RA-BN Lattice Algorithm

This section defines an algorithm for generating the Joint RA-BN lattice of neutral system general and specific graphs.

#### 4.6.1. Procedure to Generate the RA Neutral System General and Specific Graphs from a Single Rho Graph

This is done in three steps: in Step 1, generate the most complex set of specific graphs that correspond to the Rho graph; in Step 2, generate all their less complex specific graph descendants; in Step 3, specific graphs are collected together in general graphs. 

**Figure 30:**
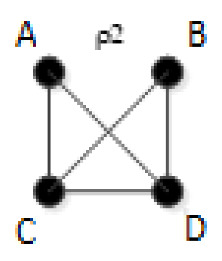
Example, Rho 2.

Step 1 begins with (Step 1.1) labeling the Rho graph, as shown in Figure 30. The most complex specific graph that corresponds to this labeled Rho graph is obtained (Step 1.2) by representing each clique with a single relation encompassing all the variables in the clique and then joining these relations with a “:”. For example, in Figure 30, A, B, and C are in a clique, i.e., are fully linked to one another and this is also the case for B, C, and D, but A and B are not linked. The resulting specific graph is ABC:BCD, which is encompassed in RA general graph G7. Next (Step 1.3), permute all the variables in this specific graph, which generates the other five specific graphs that are encompassed within G7, as shown in RA lattice of Figure 4. 

Step 2 then generates the simpler RA representations of G7 that map to Rho2, namely the specific graphs that are encompassed within the RA general graphs G8 and G9. Klir [7] (p. 231) details the procedure for this step. In Step 3, specific graphs with the same independence structure are then collected together in general graph equivalence classes. Doing this for Rho 2 results in general graphs G7, G8 and G9 and their specific graphs as shown in Figure 4. 

#### 4.6.2. Procedure to Generate the BN Neutral System General and Specific Graphs from a Single Rho Graph

**Figure 31:**
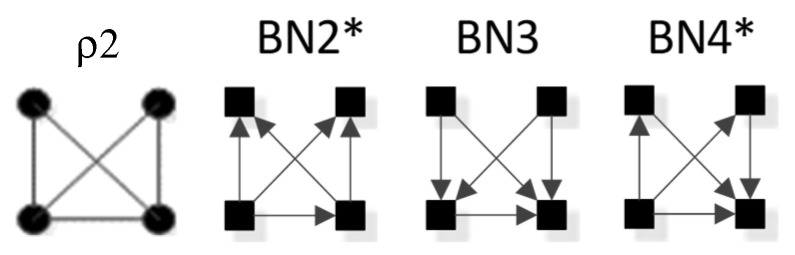
Rho 2 example, with associated BNs general graphs.

In contrast to RA graphs, BNs are just Rho graphs with directions added to edges, as shown in Figure 31. To generate all BN specific graphs for a given Rho graph, simply permute all possible edge directions and variable combinations, and follow the BN neutral system general and specific graph procedure outlined above in Section 3.2.6. Essentially, the process entails discarding redundant specific graphs and graphs with cycles from all these permutations, and collecting together BN specific graphs with unique independence structures into a general graph.

#### 4.6.3. Generating the Joint RA-BN General and Specific Graph Lattice

The following provides a general algorithm to generate the joint RA-BN lattice of neutral system general and specific graphs for any number of variables from some specific starting graph, either downwards or upwards.

1. Identify a starting Rho graph
2. Generate all possible RA and BN specific graphs for the given Rho graph.
  - For RA, follow the procedure detailed in the prior Section 4.6.1.
  - For BN, follow the procedure detailed in the prior Section 4.6.2.
  - Organize all RA and BN general graph equivalence classes into three categories: RA graphs with loops, BN graphs with V-Structures, and equivalent RA-BN graphs containing no loops or V-structures.
3. If searching the lattice upward, add an edge to the prior Rho graph. If searching the lattice downward, delete an edge from the prior Rho graph.
4. Repeat steps 2 and 3 until the top or bottom of the lattice is reached.

Consider for example the results of the RA and BN procedures for Rho 2. Organizing these results via step 2c gives the following six general structures: G8 and G9 for RA graphs with loops, BN3 and BN4* for BN graphs with V-structures, and G7 and BN2* for equivalent RA-BN graphs. Specific structures can be simply obtained from these general structures by listing all permutations of variable labels. Following these procedures for any number of variables will result in the exhaustive, non-redundant, lattice of joint RA-BN neutral system general and specific graphs.

## 5. Comparing RA and BN Directed System Graphs

**Figure 32:**
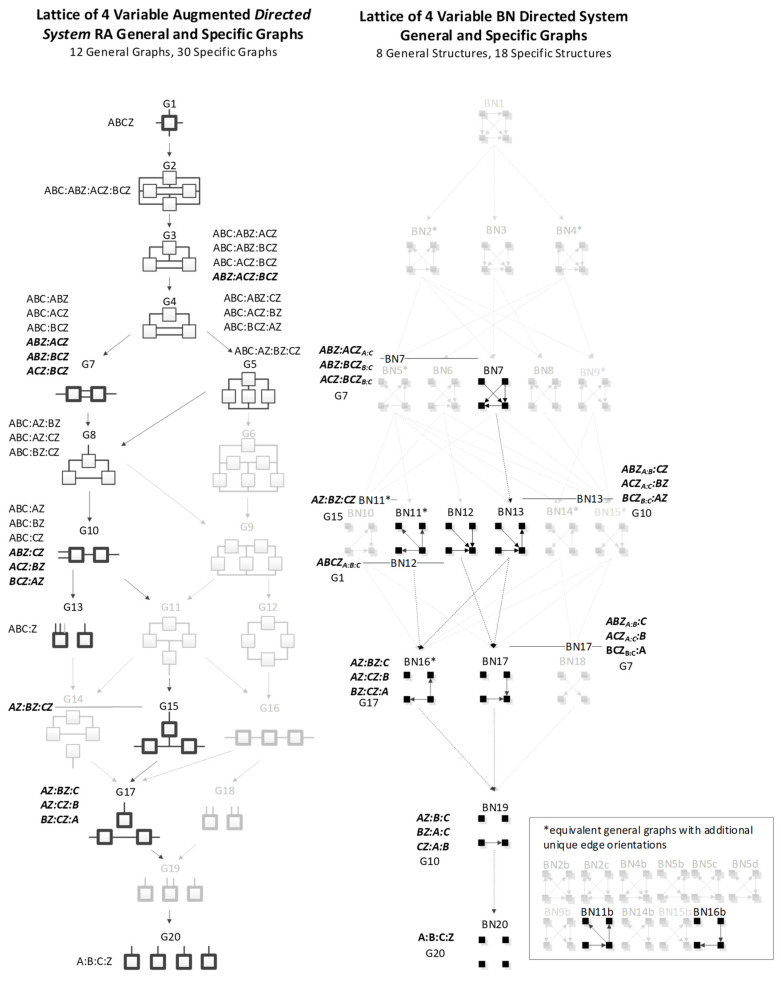
Comparison of RA augmented directed system lattice to BN directed system lattice.

Figure 32 shows side-by-side for comparison the RA augmented directed system lattice from Figure 7 and the BN directed system lattice from Figure 16. To the left or right of each BN directed system general graph is the equivalent RA directed system general graph. For example, BN7 is equivalent to RA general graph G7. Equivalence in this context is in terms statistical equivalence of prediction results given data. Two directed system general graphs are equivalent if they predict the DV (Z) in the same way. Each of the BN directed system general graphs in the lattice is equivalent to an RA general graph in the augmented RA directed system general graph lattice. In addition, the RA directed system lattice includes additional predictive graphs, those with loops that are not found in the BN lattice. Thus, restricting BN directed systems to those where the DV is not a parent in a V-structure, the RA augmented directed system lattice fully encompasses the BN directed system lattice and offers additional predictive graphs. 

Table 6 shows all BN directed system general graphs and their RA equivalents as well as specific graph examples with their associated probability distributions. In these probability distributions, only the terms used to predict the DV (Z) are highlighted in black; non-predictive terms are greyed. All equivalences necessarily involve loopless RA models; half of these involve RA graphs in the standard directed system lattice, where every model has an IV component, and the other half involve graphs in the augmentation of this lattice. Prior to development of the BN directed system lattice in this paper, the RA directed system lattice did not include naïve Bayes equivalent graphs, e.g., G15 and G17, and the naïve Bayes-like graph, G10. The development of the BN directed system lattice in this paper in part inspired the augmentation of the standard RA directed system lattice to include naïve Bayes type graphs.

However, as pointed out above, the BN directed system lattice developed in this paper was constrained to disallow any DV that is a parent node within a V-structure. If this constraint were to be relaxed to allow DVs that are parent nodes in V-structures, then there are BN predictive models that give different analytical results than RA predictive models. Therefore, the BN directed system lattice developed in this paper is preliminary and incomplete.

**Figure 33:**
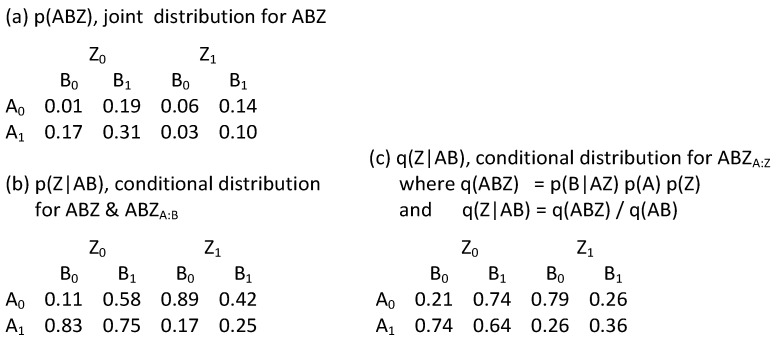
BN Directed System Prediction Example.

To illustrate this point, consider BN17 from Figure 16 with its specific graph ABZ_A:B_:C, and removing variable “C” for simplicity resulting in ABZ_A:B_ with edge orientations A->Z<-B and with probability distribution p(Z|AB)p(A)p(B). Here, Z is the DV and is the child node within the V-structure and is thus included within the BN directed system lattice developed in this paper. This graph is equivalent in terms of prediction to RA directed system graph G7 with specific graph ABC:ABZ. In contrast, consider BN17 with its specific graph ABZ_A:Z_:C. Again, for simplicity and comparability, removing variable “C” results in ABZ_A:Z_ with edge orientations A->B<-Z and with probability distribution p(B|AZ)p(A)p(Z). Here, Z is the DV, and is a *parent* node within the V-structure; therefore, this specific graph was not considered in the BN directed system lattice developed in this paper. However, the predicting components within the probability distribution are different and thus will result in a different statistical result. The differences between ABZ_A:B_ and ABZ_A:Z_ are illustrated in Figure 33, in which a hypothetical joint probability distribution p(ABZ), shown in (a), yields a conditional distribution p(Z|AB) for RA model ABZ and BN model ABZ_A:B_, shown in (b), that is different from the conditional distribution q(Z|AB) for BN model ABZ_A:Z_, shown in (c). ABZ_A:Z_ is an unconventional BN model in its choice of the parent node Z as the DV. These non-conventional BN models are not considered in this paper, but are a promising topic for future research that will extend the work reported here. 

## 6. Discussion

### 6.1. Neutral Systems

This paper builds on the RA work of Harris and Zwick [33], which developed the BN neutral system general graph lattice of Figure 8, expanding it here to offer the BN neutral system specific graph lattice of Figure 14. This paper also builds on the joint RA-BN neutral system general graph lattice of Figure 29 developed in that earlier work, expanding it here to offer the joint RA-BN neutral system specific graph lattice of Figure A6. In developing these new lattices, this paper extends RA notation to encompass BN graphs (see Section 3.2.3). 

For four variables, the joint RA-BN neutral system general graph lattice increases the number of general graphs from 20 in the RA lattice and 20 in the BN lattice to 30 in the joint RA-BN lattice, and unique specific graphs from 114 in the RA lattice and 185 in the BN lattice to 238 in the joint lattice. The integration of the two lattices offers a richer and more expansive way to model and represent complex systems leveraging the V-structure unique to BN graphs and the ability accommodate loops and hypergraphs in the RA lattice. 

This paper also develops an algorithm to generate the joint RA-BN neutral system general and specific graph lattices for any number of variables in both upward and downward directions (Section 4.6). The exhaustive and non-redundant RA and BN lattices follow the more general Rho lattice. Figure A6 shows the results of this algorithm for four variables. Although this algorithm is exhaustive, it does not create a hierarchical nesting of general or specific graphs. Such nesting is a desirable feature, so future extensions of this work could enhance the algorithm by enabling it to develop sequentially with each new graph being hierarchically nested. Given data, such an extension would allow statistical significance tests to be performed at each incremental step of lattice generation. Additionally, the current algorithm produces the exhaustive lattice, but searching the exhaustive lattice to find best candidate graphs is inefficient, so algorithms to efficiently search the joint lattice for best candidate graphs would be a useful extension.

Another promising extension of this work would be to develop hybrid RA-BN general graphs [13] for neutral systems to further extend the expression of the joint RA-BN neutral system lattice developed in this paper. Such hybrid graphs could incorporate directed edges to encode BN V-structures with loops and hypergraphs found in RA. Other possible extensions of this work could explore the application of Bayesian networks to hypergraphs [56] and under appropriate conditions to certain types of cycles [57]. 

### 6.2. Directed Systems

This paper develops the RA augmented directed system lattice (Figure 7), which is an extension of the conventional RA directed system lattice (Figure 5). While the conventional RA directed system lattice encompasses all prediction graphs in the BN directed system lattice (under the restriction that DVs in BN models are not parent variables in V-structures), the RA conventional directed system lattice did not include naïve Bayes graphs. Doing so, as shown in Figure 7, increases the number of general graphs from nine in the conventional RA lattice to 12 in the augmented lattice, and the number of specific graphs from 19 to 31. The augmented RA directed system lattice thus offers more candidate graphs, and this allows for the possibility of more accurate or simpler and thus more generalizable RA prediction models. Augmentation of the conventional RA directed system lattice was inspired in part by the BN directed system lattice developed in this paper.

Future extension of this work could examine whether BN graphs with predictions equivalent to RA models but with fewer degrees of freedom than RA predictive equivalents (because of independence constraints among the IVs) offer any advantage in calculations of statistical significance. If so, such BN graphs might replace their RA equivalents in the augmented directed system RA lattice. A related statistical issue that should be explored is how to compare augmenting directed RA models whose natural reference is A:B:…, the neutral system independence reference, with conventional directed systems models whose natural reference is AB…:Z, i.e., a reference that has an IV component that joins together all IVs in a single relation. 

This paper develops the BN directed system lattice of prediction graphs for four variables (Figure 16), reducing the number of possible specific graphs from 185 in the BN neutral system lattice to 18 in the BN directed system lattice—a significant compression of the BN neutral system lattice when prediction of a single DV is the goal. This paper also shows that all of the graphs in the BN directed system lattice (where this lattice disallows graphs where the DV is a V-structure parent) are equivalent in their predictions to RA graphs, although many of them have fewer degrees freedom than their RA-equivalent counterpart. The augmented RA directed system lattice thus encompasses all of the BN directed system general graphs in terms of prediction, and offers additional predicative graphs, those including loops, that are not in the BN lattice. However, the restriction that disallows BN graphs where the DV is a V-structure parent might be relaxed, so a future extension of this work could consider expanding the BN directed system lattice to include such unusual BN predictive graphs. An additional extension could be to develop an algorithm to generate the BN directed system lattice of general and specific graphs for any number of variables allowing for efficient search of the BN lattice for graphs that uniquely predict a single DV.

## Figures and Tables

**Table 1:** RA and BN terminology.

	Our Terminology	Literature Terminology	Lattice Name, RA-Like Notation	Visuals
RA	General RA graph	G-structures [7]	G15 (Figure 1)	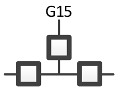
	Specific RA graph	Specific RA graph [15]	G15 (Figure 4), AD:BD:CD	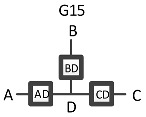
BN	General BN graph	Maximally oriented graphs, essential graphs, equivalence classes of directed acyclic graphs, partially directed graphs [29,30,31,32]	BN11* & BN11b (Figure 8)	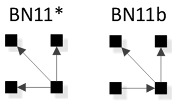
	Specific BN graph (no-V-structure)	Labeled maximally oriented graphs, essential graphs, equivalence classes of directed acyclic graphs, partially directed graphs	BN11*, BN11b (Figure 14), AD:BD:CD	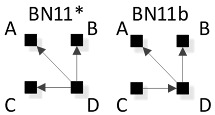
	Specific BN graph (V-structure)		BN17 (Figure 14), BCD_B:C_:A	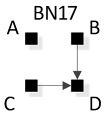

**Table 2:** Four-variable independence statements.

	Marginal Independence			Conditional Independence	
General Expression	(. ⊥ ..)	(. ⊥ ..,…)	(. ⊥ .., …, ….)	(. ⊥ .. | …)	(. ⊥ .. | …, ….)
Specific Expression 1	(A ⊥ B)	(A ⊥ B, C)	(A ⊥ B, C, D)	(A ⊥ B | C)	(A ⊥ B | C, D)
2	(A ⊥ C)	(A ⊥ B, D)	(B ⊥ A, C, D)	(A ⊥ B | D)	(A ⊥ C | B, D)
3	(A ⊥ D)	(A ⊥ C, D)	(C ⊥ A, B, D)	(A ⊥ C | D)	(A ⊥ D | B, C)
4	(B ⊥ C)	(B ⊥ A, C)	(D ⊥ A, B, C)	(B ⊥ A | C)	(B ⊥ A | C, D)
5	(B ⊥ D)	(B ⊥ A, D)		(B ⊥ A | D)	(B ⊥ C | A, D)
6	(C ⊥ D)	(B ⊥ C, D)		(B ⊥ C | D)	(B ⊥ D | A, C)
7		(C ⊥ A, B)		(C ⊥ A | B)	(C ⊥ A | B, D)
8		(C ⊥ A, D)		(C ⊥ A | D)	(C ⊥ B | A, D)
9		(C ⊥ B, D)		(C ⊥ B | D)	(C ⊥ D | A, B)
10		(D ⊥ A, B)		(D ⊥ A | B)	(D ⊥ A | B, C)
11		(D ⊥ A, C)		(D ⊥ A | C)	(D ⊥ B | A, C)
12		(D ⊥ B, C)		(D ⊥ B | C)	(D ⊥ C | A, B)

**Table 3:** Probability distribution and independencies of BN specific graph examples.

BN General Graph	Specific Graph Example		
	RA Notation	Probability Distribution	Independencies
BN1	ABCD	p(B|A)p(A)p(C|AB)p(D|ABC)	none
BN2	ACD:BCD	p(A|CD)p(C)p(B|CD)p(D|C)	(A ⊥ B | C, D)
BN3	ABCD_A:B_	p(C|AB)p(A)p(B)p(D|ABC)	(A ⊥ B)
BN4	ABCD_AC:BC_	p(A|C)p(C)p(B|C)p(D|ABC)	(A ⊥ B | C)
BN5	BCD:AD	p(A|D)p(D)p(B|CD)p(C|D)	(A ⊥ B, C | D)
BN6	ABCD_BC:A_	p(B|C)p(C)p(D|ABC)p(A)	(A ⊥ B, C)
BN7	BCD:ABD_A:B_	p(C|BD)p(B)p(D|AB)p(A)	(A ⊥ B), (A ⊥ C | B, D)
BN8	ACD_C:D_:BCD_C:D_	p(A|CD)p(C)p(D)p(B|CD)	(C ⊥ D), (A ⊥ B | C, D)
BN9	ABD:ABC_AC:BC_	p(A|C)p(C)p(B|C)p(D|AB)	(A ⊥ B | C), (C ⊥ D | A, B)
BN10	BCD:A	p(B|C)p(C)p(D|BC)	(A ⊥ B, C, D)
BN11	AD:BD:CD	p(A|D)p(D)p(B|D)p(C|D)	(A ⊥ B, C | D), (B ⊥C | D)
BN12	ABCD_A:B:C_	p(D|ABC)p(A)p(B)p(C)	(A ⊥ B, C), (B ⊥ C)
BN13	ACD_A:C_:BD	p(B|D)p(D|AC)p(A)p(C)	(A ⊥ C), (B ⊥ A, C |D)
BN14	AD:BC:BD	p(A|D)p(D)p(B|D)p(C|B)	(A ⊥ B | D), (C ⊥ A, D | B)
BN15	ABD_A:B_:BC	p(C|B)p(B)p(D|AB)p(A)	(A ⊥ B, C), (C ⊥ D | A, B)
BN16	BD:CD:A	p(B|D)p(D)p(C|D)p(A)	(B ⊥ C | D), (A ⊥ B, C, D)
BN17	BCD_B:C_:A	p(D|BC)p(B)p(C)p(A)	(B ⊥ C), (A ⊥ B, C, D)
BN18	AD:BC	p(C|B)p(B)p(D|A)p(A)	( A, D ⊥ B, C)
BN19	CD:A:B	p(D|C)p(C)p(A)p(B)	(B ⊥ C, D), (A ⊥ B, C, D)
BN20	A:B:C:D	p(A)p(B)p(C)p(D)	(A ⊥ B, C, D), (B ⊥ C, D), (C ⊥ D)

**Table 4:** BN directed system graphs.

BN General Graph	Predictively Equivalent Simpler Graph	Specific Graph Example RA Notation	Specific Graph Example Probability Distribution
BN1	BN12	ABCZ	p(Z|ABC)p(C|AB)p(B|A)p(A)
BN2	BN7 BN17	ABZ:BCZ ABC:BCZ	p(C|BZ)p(Z|AB)p(A|B)p(B) p(Z|BC)p(B|CA)p(A|C)p(C)
BN3	BN12	ABCZ_A:B_	p(Z|ABC)p(C|AB)p(A)p(B)
BN4	BN12	ABCZ_AC:BC_	p(Z|ABC)p(A|C)p(B|C)p(C)
BN5	BN13 BN19	ACZ:BZ ABC:CZ	p(Z|AC)p(B|Z)p(C|A)p(A) p(Z|C)p(B|CA)p(C|A)p(A)
BN6	BN12	ABCZ_BC:A_	p(Z|ABC)p(B|C)p(C)p(A)
BN7	BN17	BCZ:ABZ_A:B_ BCZ:ABC_A:C_	p(C|BZ)p(Z|AB)p(B)p(A) p(Z|BC)p(B|CA)p(C)p(A)
BN8	BN17	ABC_B:C_:BCZ_B:C_	p(Z|BC)p(A|BC)p(B)p(C)
BN9	BN17	BCZ:ABC_AB:AC_	p(Z|BC)p(B|A)p(C|A)p(A)
BN10	BN17	BCZ:A	p(Z|BC)p(B|C)p(C)p(A)
BN11	BN19	AZ:BZ:CZ AC:BC:CZ	p(A|Z)p(B|Z)p(C|Z)p(Z) p(Z|C)p(B|C)p(C|A)p(A)
BN12		ABCZ_A:B:C_	p(Z|ABC)p(A)p(B)p(C)
BN13	BN19	ACZ_A:C_:BZ ABC_A:B_:CZ	p(Z|AC)p(B|Z)p(A)p(C) p(Z|C)p(C|AB)p(A)p(B)
BN14	BN16 BN19	AB:BZ:CZ AB:BC:CZ	p(Z|B)p(C|Z)p(B|A)p(A) p(Z|C)p(B|A)p(A)p(C|B)
BN15	BN17 BN19	BCZ_B:C_:AB ABC_A:C_:CZ	p(Z|BC)p(A|B)p(B)p(C) p(Z|C)p(B|CA)p(A)p(C)
BN16	BN19	BZ:CZ:A BC:CZ:A	p(B|Z)p(C|Z)p(Z)p(A) p(Z|C)p(C|B)p(B)
BN17		BCZ_B:C_:A	p(Z|BC)p(B)p(C)p(A)
BN18	BN19	AB:CZ	p(Z|C)p(B|A)p(A)p(C)
BN19		CZ:A:B	p(Z|C)p(C)p(A)p(B)
BN20		A:B:C:Z	p(Z)p(A)p(B)p(C)

**Table 5:** Equivalent Rho, RA and BN neutral system general graphs.

Rho Graph	RA General Graph	BN General Graph	Specific Graph Example (RA Notation)	Independencies
ρ1	G1	BN1	ABCD	no independencies
ρ2	G7	BN2*	ACD:BCD	(A ⊥ B | C, D)
ρ3	G10	BN5*	BCD:AD	(A ⊥ B, C | D)
ρ5	G13	BN10	BCD:A	(A ⊥ B, C, D)
ρ6	G15	BN11*	AD:BD:CD	(A ⊥ B, C | D), (B ⊥ C | D)
ρ7	G16	BN14*	AD:BC:BD	(A ⊥ B | D), (C ⊥ A, D | B)
ρ8	G17	BN16*	BD:CD:A	(B ⊥ C | D), (A ⊥ B, C, D)
ρ9	G18	BN18	AD:BC	(A, D ⊥ B, C)
ρ10	G19	BN19	CD:A:B	(B ⊥ C, D), (A ⊥ B, C, D)
ρ11	G20	BN20	A:B:C:D	(A ⊥ B, C, D), (B ⊥ C, D), (C ⊥ D)

**Table 6:** BN directed system graphs and RA equivalent example.

BN General Graph	BN Specific Graph Example RA Notation	BN Specific Graph Example Probability Distribution	Equivalent RA Graph	Equivalent RA Graph Notation	Equivalent RA Graph Probability Distribution
BN7	BCZ:ABZ_A:B_	p(C|BZ)p(Z|AB)p(B)p(A)	G7 (augmentation)	BCZ:ABZ	p(C|BZ)p(Z|AB)p(B|A)p(A)
BN11	AZ:BZ:CZ	p(A|Z)p(B|Z)p(C|Z)p(Z)	G15 (augmentation)	AZ:BZ:CZ	p(A|Z)p(B|Z)p(C|Z)p(Z)
BN12	ABCZ_A:B:C_	p(Z|ABC)p(A)p(B)p(C)	G1	ABCZ	p(Z|ABC)p(ABC)
BN13	ACZ_A:C_:BZ	p(Z|AC)p(B|Z)p(A)p(C)	G10 (augmentation)	ACZ:BZ	p(Z|AC)p(B|Z)p(C|A)p(A)
BN16	BZ:CZ:A	p(B|Z)p(C|Z)p(Z)p(A)	G17 (augmentation)	BZ:CZ:A	p(B|Z)p(C|Z)p(Z)p(A)
BN17	BCZ_B:C_:A	p(Z|BC)p(B)p(C)p(A)	G7	ABC:BCZ	p(Z|BC)p(B|CA)p(A|C)p(C)
BN19	CZ:A:B	p(Z|C)p(C)p(A)p(B)	G10	ABC:CZ	p(Z|C)p(ABC)
BN20	A:B:C:Z	p(Z)p(A)p(B)p(C)	G13	ABC:Z	p(Z)p(ABC)

**Figure A1:**
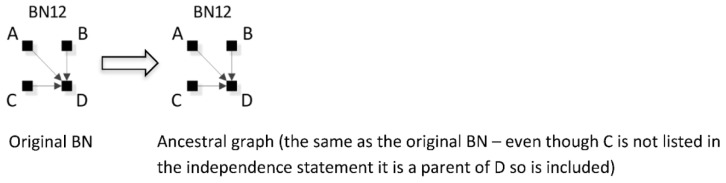
Step 2, create the ancestral graph.

**Figure A2:**
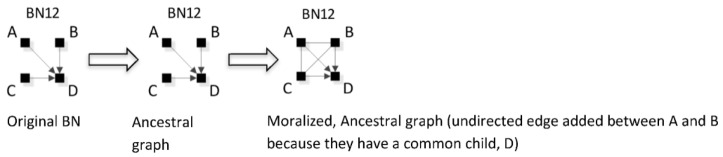
Step 3, moralize the ancestral graph.

**Figure A3:**
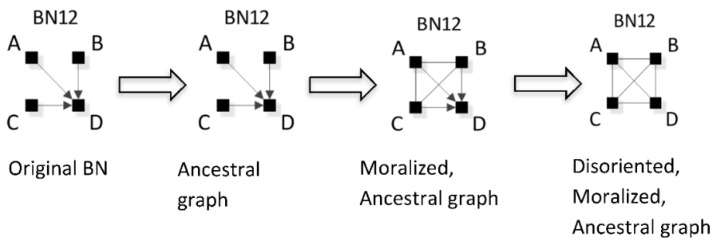
Step 4, disorient the moralized, ancestral graph.

**Figure A4:**
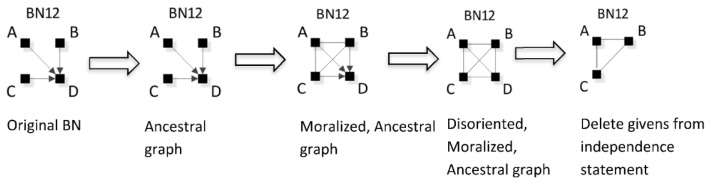
Step 5, delete the givens.

**Figure A5:**
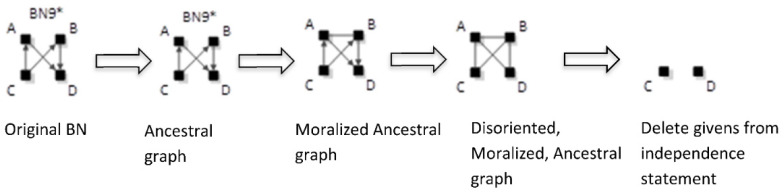
Example, full D-separation procedure for independence statement (C ⊥ D|A, B).

## Appendix A

The contents of this Appendix are the work of other researchers and are included here to allow this paper to be understood in a self-contained way.

### Appendix A.1. RA Loop Detection Procedure

In the RA graphs of Figure 1 and Figure 4, graphs that do not have loops are highlighted in bold, while graphs that have loops are non-bold. Graphs without loops are fitted algebraically, whereas graphs with loops are fitted using iterative proportional fitting. To determine if a graph has a loop, the following procedure is performed:

Given the set of relations of a specific graph:

1. Remove all variables that are unique to any individual relation
2. Remove any relation that is equal to or embedded in any other relation of the (remaining) set
3. Repeat 1 and 2 until either
  - No variables remain, in which case there are no loops, or
  - The remainder is unalterable by steps 1 or 2, in which case there are loops

For example, in Figure 4, graph G7, illustrated by specific graph ABC:ABD does not have a loop. Krippendorff’s loop detection algorithm [11] produces the following results. First, removing variables unique to both ABC and ABD removes C from ABC and D from ABD. What remains is AB:AB, for which the second AB is redundant and thus removed, leaving AB. Then, removing the variables unique to AB removes both A and B, leaving the null set, and thus this specific graph does not contain a loop.

By contrast, in Figure 4, graph G8, illustrated by specific graph ABC:AD:BD, does have a loop. Krippendorff’s loop detection algorithm [11] produces the following results. First, removing variables unique to one relation removes C from ABC. What remains is AB:AD:BD. There is no redundant relation, i.e., no relation that is repeated or embedded in another relation. There are also no variables unique to one relation. Therefore, nothing further can be removed; because the remaining set is unalterable, the specific graph has a loop.

### Appendix A.2. D-Separation Procedure

The following provides the procedure for determining all independencies for a BN [55]

Step 1. List all possible independence statements for a given BN.
Step 2. For each independence statement, construct the ‘ancestral graph’ [58] for the variables mentioned in the independence statement.
Step 3. ‘Moralize’ the ancestral graph by adding an undirected edge between two nodes if they have a common child.
Step 4. ‘Disorient’ the moralized, ancestral graph, by making all edges undirected.
Step 5. Delete the givens (nodes) and any of their edges from the independence statement being tested.
Step 6. Read the answer to the independence statement question from the remaining graph, if the variables are disconnected in the remaining graph, the answer to the independence statement is in the affirmative.

The following provides examples of the D-separation procedure for BN12 and BN9*:

Example 1

Step 1. List all possible independence statements for a given BN. For four variables, Table 2 is the complete list.

Step 2. For each independence statement, construct the ‘ancestral graph’ [58] for the variables mentioned in the independence statement.

An ancestral graph of the probability expression includes all nodes listed in the independence statement that is being tested and all parents, grandparents, great-grandparents, etc., of those nodes.

BN12 and the independence statement (A ⊥ B | D) will be used as an example throughout the remaining procedure (Steps 2–5, Figure A1, Figure A2, Figure A3, Figure A4 and Figure A5).

Step 3. ‘Moralize’ the ancestral graph by adding an undirected edge between two nodes if they have a common child.

Step 4. ‘Disorient’ the moralized ancestral graph by making all edges undirected.

Step 5. Delete the givens from and any of their edges. In the continuing example, D is the ‘given’ (A ⊥ B | D), thus D and its connected edges to A, B, and C are removed.

Step 6. Read the answer to the independence statement question from the remaining graph, if the variables are disconnected in the remaining graph, the answer to the independence statement is in the affirmative. In this example, the independence statement being tested is the assertion that A and B are independent given D. This assertion is false because A and B are connected in the remaining graph; thus, they are not *conditionally* independent given D.

Example 2

Consider a second example, using BN9*. The independence statement being tested here is the assertion: C independent of D given A and B (C ⊥ D|A, B). Figure A5 shows all steps in the procedure for this example, affirming C and D are indeed independent given A and B.
